# A double-blinded randomised dietary supplement crossover trial design to investigate the short-term influence of medium chain fatty acid (MCT) supplement on canine idiopathic epilepsy: study protocol

**DOI:** 10.1186/s12917-019-1915-8

**Published:** 2019-05-30

**Authors:** Benjamin Andreas Berk, Rowena Mary Anne Packer, Tsz Hong Law, Annette Wessmann, Andrea Bathen-Nöthen, Tarja Susanna Jokinen, Anna Knebel, Andrea Tipold, Ludovic Pelligand, Holger Andreas Volk

**Affiliations:** 1 Department of Clinical Science and Services (CSS), Royal Veterinary College, Hatfield, UK; 2 Tierarztpraxis Strassenheim: BrainCheck.Pet, Ortsstraße, Mannheim, Germany; 3 Pride Veterinary Centre, Neurology/Neurosurgery Service, Riverside Road, Pride Park, Derby, UK; 4 Tierarztpraxis, Dr. A. Bathen-Nöthen, Hatzfeldstraße, Cologne, Germany; 5 Department of Equine and Small Animal Medicine, Faculty of Veterinary Medicine, Helsinki, Finland; 6 Klinik für Kleintiere, Stiftung Tierärztliche Hochschule Hannover, Bünteweg, Hanover, Germany; 7 Department of Comparative Biomedical Sciences (CBS), Royal Veterinary College, Hatfield, UK

**Keywords:** Veterinary neurology, Epilepsy, Diet, Medium-chain triglyceride, Medium-chain fatty acids, Seizure

## Abstract

**Background:**

Epilepsy is the most common brain disease in dogs. Recently, diets have been reported to have a positive impact on seizure activity and behaviour in various species including dogs with idiopathic epilepsy (IE). Historically, classic high fat ketogenic diets (KD) and medium chain triglycerides (MCT) KD have been successfully used to manage drug-resistant epilepsy. Similarly, an MCT enriched diet has been shown to improve seizure control and behavioural comorbidities in some dogs with IE. However, it is unknown whether an MCT dietary supplement (DS) may provide similar positive effects.

**Methods:**

A 6-month prospective, randomised, double-blinded, placebo-controlled, crossover, multicentre dietary trial is designed comparing a 9% metabolic energy based calculated medium-chain triglyceride (MCT) oil supplement to a conventional ‘control’ DS. Only dogs which will have an International Veterinary Epilepsy Task Force Tier II level like diagnosis of IE which satisfied the following inclusion criteria are included: age between 6 months and ≤ 12 years; weighing between 4 and ≤ 65 kg; unremarkable interictal neurological examinations; no clinically significant findings on routine laboratory diagnostics; unremarkable brain MRI scan; have had at least 3 seizures in the previous 3 months prior to enrolment; treated with at least one ASD and being classified as resistant. All dogs are fed initially for 90 ± 2 days with either the control oil or the MCT oil alongside their normal diet, followed by 97 ± 2 days with the other supplement including a 7-day washout period. Overall, the aim is to recruit thirty-six patients at five different centres and to investigate the effect of MCTs as DS on seizure activity, tolerability, behavioural comorbidities and quality of life (QoL).

**Discussion:**

Dietary interventions are rarely studied in a standardised form in veterinary medicine. The background diet, the cohort of animals and ASD received is standardised in this prospective diet trial to ensure representative data about the potential effect of MCT DS. If the study data confirms former findings, this would provide further evidence for the efficacy of MCTs as a management option for canine epilepsy. This publication should offer a repository of trial conditions and variable description with forecasted statistical analysis.

**Electronic supplementary material:**

The online version of this article (10.1186/s12917-019-1915-8) contains supplementary material, which is available to authorized users.

## Background

Epilepsy, defined by the disposition to having seizures, is the most common chronic neurological disease in humans and dogs [1, 2]. Within the UK alone, epidemiological studies have revealed that an estimated 0.6% of dogs are affected by idiopathic epilepsy (IE) [3]. Epilepsy is a major risk to health and welfare in dogs. This neurological disorder has been associated with physical, cognitive and neurobehavioural comorbidities and increased risk of premature death [4].

Currently, pharmacotherapy with seizure suppressing drugs (“anti-epileptic drugs ”or “anti-seizure drugs” (ASD)) represents the most important form of treatment in veterinary medicine. Epileptic patients are usually treated on a chronic, often life-long basis [4]. However, permanent ASD administration is characterised by determining the fine balance between benefits and disadvantages of ASDs. The seizure-suppressing effects of ASDs might be outweighed by unfavourable side effects including polyphagia, polydipsia, polyuria, restlessness, lethargy or ataxia [5]. Owners of dogs with IE are often worried about the level of sedation and ataxia induced by ASDs, their dog’s seizure frequency, and polypharmacy affecting their own and their pets’ quality of life (QoL) [6].

Furthermore, despite well-established ASD therapies, over two thirds of dogs with epilepsy continue to experience seizures in the long-term and fail to achieve total epilepsy control [7–9]. Up to 30% of these dogs treated with two or more ASDs continue to experience seizures with a less than 50% reduction in seizure frequency [8, 10–12]. This emphasises the need for new therapeutic interventions to improve the QoL of dogs with IE [7, 13–15].

Various diets, especially ketogenic diets (KD), have been shown to reduce frequency and severity of seizures in humans with epilepsy and in rodent models of epilepsy [14, 16–21]. Lifestyle variables, such as the diet of dogs with IE, have also been recognised both anecdotally and in published literature as having an impact upon seizure activity and behaviour [22–25]. For example, a trial of eight dogs with epilepsy treated with an exclusion diet was reported in 2004 with seven dogs achieving a reduction in seizure frequency [26]. Unfortunately, the exact mechanism leading to the anti-epileptic and/or anti-seizure properties of KDs has not yet been fully elucidated, highlighting the need for more research, especially in veterinary medicine, to confirm the effects of diet in dogs with IE.

The ketogenic diet was first used in human epilepsy to mimic the metabolic state of fasting, as fasting was shown to have anti-seizure effects [27]. Under conditions of low carbohydrate intake, such as fasting, the liver metabolises triglycerides and releases fatty acids and subsequent ketone bodies into blood circulation, a process known as ketosis [28]. The ketone bodies replace glucose as the main source of energy in the brain [29]. A rise in brain ketones has been suggested to evoke anti-epileptic properties [30]. The ketone body Beta-hydroxybutric acid (BHB) has been shown to reduce seizure-like neuronal activity and is thought to be mediated via K_ATP_ channel activity and inhibiting GABA_B_ signalling in a Drosophila in-vitro model [31]. Similarly, intraperitoneal injection of BHB led to increased BHB serum concentrations and prolonged onset of seizures in a rat seizure model accompanied with neuroprotective effects observed in histopathology [32]. Other, newer in-vitro studies assumed BHB as having neuroprotective function by direct gene regulation and promotion of brain-derived neurotrophic factor (BDNF) expression [33]. Apart from BHB, other in-vitro and in-vivo studies in rodent epilepsy models on other ketone bodies, such as acetone or acetoacetate, also indicated anticonvulsant action, ion channel interaction and potential synergistic role in ketogenic diets [34–38]. Many studies have reported the anti-seizure effects of different ketogenic diets and as such a myriad of hypotheses have been generated for the mechanisms of action [39–42]. Proposed mechanisms behind this dietary intervention include preventing neuronal hyperexcitability, direct inhibition of ion channels, influencing mitochondrial functions and altering amino acid metabolism to influence specific inhibitory neurotransmitter production [43–45].

At present, it is unclear whether the original KD would prove to be an effective anti-epileptic management option in dogs. To improve palatability and ketonemia, a variant of ‘classic‘KD was introduced in 1971 by Huttenlocher [46]. This subtype utilises medium chain triglycerides (MCT) as fat resource found as being more ketogenic than long chain triglycerides (LCT) [47]. Up to date, many research groups found that MCT-KD had also good anti- seizure properties [16, 41, 48]. As dogs have a higher capacity for ketone body utilisation, it is hard to induce and/or maintain clinically effective levels of ketosis and anticonvulsant efficacy in dogs comparable to human medicine [49]. In contrast, MCTs are considered also as being more ketogenic in dogs due to subsequent transportation without pancreatic response and primarily metabolization into ketone bodies in the liver [39, 48–50]. MCT-KD came therefrom more into research focus as the ketogenic diet for dogs [23, 24, 49, 51].

The main constituent of MCT-KD are normally octanoic (C8, caprylic) and decanoic (C10, capric) triglycerides (TAGs) [52, 53]. A recent in-vitro study has revealed decanoic acid acts as a non-competitive AMPA receptor antagonist that results in direct inhibition of excitatory neurotransmission, and thus exerts an anticonvulsant effect [54]. In comparison, octanoic acid alone revealed no anti-epileptic effects in in-vitro studies [55], but enhanced anticonvulsive properties in mice, when orally co- administered with C10 [56]. Ongoing research focusing on in vitro and rodent models also highlighted the importance of medium chain fatty acids with specific branching as anticonvulsant [57, 58].

In dogs, consumption of an MCT-enriched diet (10% of total formula calories with C8, C10, C12) leads to significant elevation in BHB concentration and improved seizure control [24, 45, 59–61]. MCT intake also appears to be linked to a significant improvement in attention- deficit hyperactivity disorder (ADHD) -related behaviour and fear/anxiety in dogs with epilepsy [23] and to have cognition-improving effects in aged dogs [61]. Recent research confirmed the observed benefits from MCT enriched kibble diet in aged dogs with cognitive dysfunction syndrome (CDS) in a prospective double blinded placebo controlled study design [62].

In conclusion, albeit past research provides a solid base, the exact metabolic mechanisms governing anti- seizure effects from BHB and/or MCT remain elusive, not only in humans, but especially in dogs. Whether MCTs can be an effective anti-seizure agent and show neuroprotective and anxiolytic effects in dogs with IE requires further investigation.

We hypothesise MCTs administered as a daily supplement will reduce seizure frequency and severity, reduce the level of ASD side effects and positively influence behavioural comorbidities of epilepsy in dogs. The primary aims of this clinical study are to investigate the short- term influence of MCTs given as a DS on seizure occurrence, disease progression, drug-response, behaviour and QoL of dogs with IE. Twelve weeks of feeding is considered the minimal duration to allow short-term evaluation of efficacy and safety for diets [24, 59]. Using the same test system as in our former study which for the first time demonstrated the efficacy of an MCT- enriched kibble diet [24] will help us draw comparisons between studies and provide results in a reasonable time frame.

The rationale of the current study is not only to corroborate the former results [4, 63] using a supplement rather than using a complete manufactured diet, but also to investigate the broader effects of MCTs on epilepsy and its comorbidities including their effects on cognition and behavioural comorbidities. This study should provide further information about the role of MCT ingestion and its importance in canine epilepsy management [48, 49].

## Methods and study design

### Recruitment

Participants are informed about this trial through different media in the UK, Germany, Austria, Switzerland and Finland. For advertisement, individual flyers and websites are made supporting the recruitment process. Epilepsy phenotype is determined using previous medical history and a standardised clinical history questionnaire (Additional file 1). Throughout this study, all dogs are recruited by a two-step screening system. Initially, all interested owners are asked to complete an online pre-study questionnaire capturing important data about the signalment, history, clinical appearance, diagnostics and treatment of their dog’s IE. All applications are individually screened and selected in batches based on defined criteria and details then send to each study site.

### Study design

**Fig. 1:**
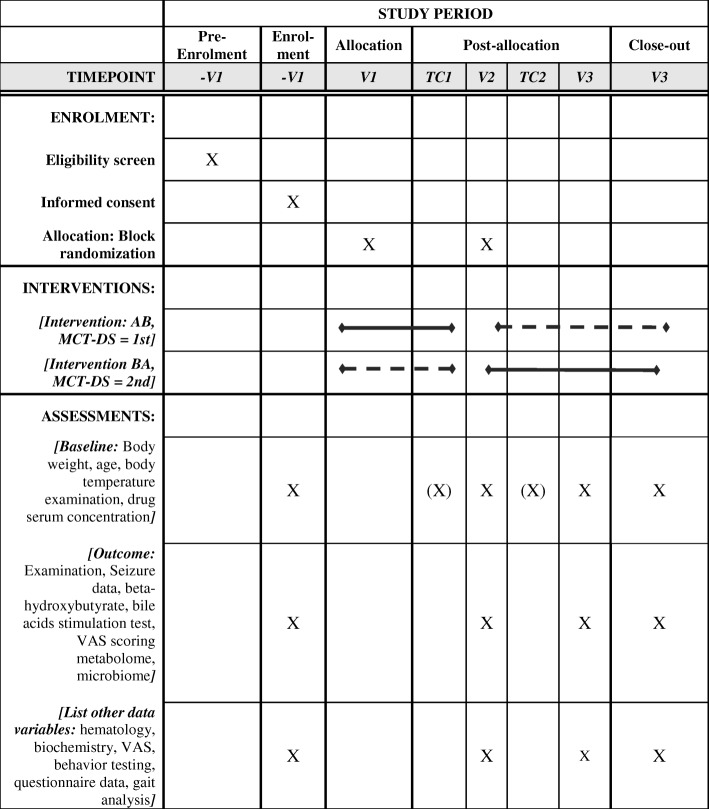
SPIRIT aligned schedule of enrolment, interventions, and assessments

**Fig. 2:**
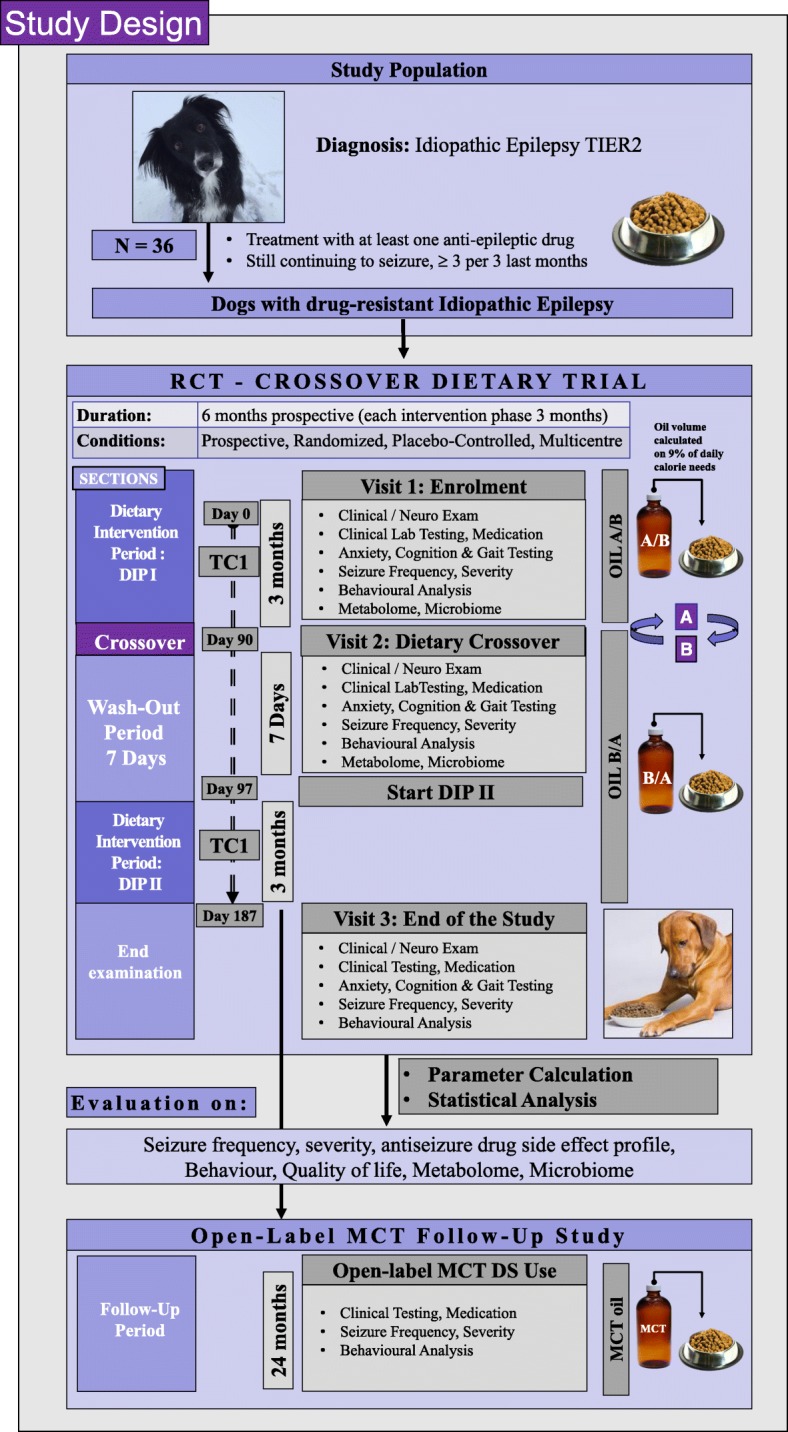
Study Design A 6-month randomised, double-blinded, prospective, multicentre, dietary crossover trial is designed to compare two oil dietary supplements (MCT oil, Control oil (=olive oil)). Both oils are given as 9% of their daily metabolic energy requirements. All dogs are aimed to be fed initially for 90+/− 2 days with either the control dietary supplement or the MCT dietary supplement alongside their normal diet followed by 97+/− 2 days (including initial one-week wash-out period, 7 days) with the other supplement alongside their normal diet. Another follow-up study about the long-term effects of MCT oil as an open-label use is subsequently planned after the individual patient has completed the main clinical trial

A 6-month randomised, double-blinded, prospective, multicentre, dietary crossover trial is designed to compare two oil-DSs (MCT oil, control oil (olive oil)) in dogs with IE (Figs. 1 and 2). The experimental unit is each individual dog. Law et al., 2015 demonstrated a significant difference in 21 dogs completing the study, with a drop-out rate of approximately 30%. In this study, we aim to enrol a total of thirty-six (36) dogs between five different study sites in three different countries in Europe (UK: Royal Veterinary College and, Pride Veterinary Centre, Germany: Tierarztpraxis Strassenheim, Tierarztpraxis Bathen-Nöthen and, Tierärztliche Hochschule Hanover, Finland: University Helsinki).

The monitoring site for this trial is the Clinical Investigation Centre (CIC) of the Royal Veterinary College (RVC), which oversees the logistical progression of the study under the supervision of the Study Monitor and leader. This study is conducted in accordance with veterinary GCP guidelines ((i) International Cooperation on Harmonisation of Technical Requirements for Registration of Veterinary Products (VICH) GL9 Good Clinical Practices (July, 2001), (ii) The European Agency for the Evaluation of Medicinal Products (EMEA) Guideline on statistical principles for veterinary clinical trials, EMEA/CVMP/816/00-FINAL (June 5, 2002)). The study is approved by the Clinical Research Ethical Review Board (URN 2016 1558).

Each dog is randomly assigned using block randomisation to begin their first dietary period with either MCT or the placebo DS. The owner and investigators will not be informed of diet group assignment for each dog. All recruited dogs will be fed initially for 90 ± 2 days with either the control DS or the MCT DS alongside their normal diet, followed by 97 ± 2 days with the other supplement alongside their normal diet. The second feeding period includes the first week as a wash out period, which will not be considered in the statistical analysis. Each dog is evaluated on-site at the enrolment visit (−V1), and at the end of each 90 ± 2-day or 2nd 97 ± 2 dietary period (V2 = 90 ± 2 days, V3 = 187 ± 2 days). Between on-site visits, owners will be contacted by phone to check for any difficulties arisen during the study period and to ensure compliance (TC1 = 45 ± 2, TC2 = 142 ± 2 days).

For all trial participants, the dog should be fed twice daily with their regular diet, with the half amount of the total daily oil requirement. A total of 9% of the daily metabolic energy per day is from the oil. ASD treatment will not be withheld at any point nor changed in this study, however, owners will be allowed to stop their participation in the trial at any time point, as requested by the Clinical Research Ethical Review Board. Dogs that have been receiving other oils or oil type DS previously can take part in this study when there has been a pre-wash out period of at least 14 days before visit 1. All owners are asked to keep the diet and drug treatment constant throughout the study period and to document any adverse events.

The ‘Animal Research: Reporting *in-Vivo* Experiments’ (ARRIVE) [64] and the Schedule Of Enrolment, Interventions, And Assessments (SPIRIT) reporting guidelines [65] have been considered and integrated into this study protocol were applicable (see also Additional file 2) .

### Investigational animals – study phenotype

Only dogs that satisfy all of the inclusion criteria and to whom none of the exclusion criteria apply will be enrolled and receive at enrolment a unique study number for identification purposes. All owners must give written informed consent to participate in the study and agree to fulfil all requirements of the study. All mixed or pure-breed dogs participating in this study are privately owned. The pet dogs should be at least 6 months of age on Day 0 and weigh at least 4 kg. To be recruited, dogs must be in general good health and have an unremarkable interictal neurological examination for a dog on ASD treatment. Additionally, each dog must have met in general the International Veterinary Epilepsy Task Force (IVETF) tier II confidence level diagnosis of IE [4]. The current IVETF statement is, however, not yet clear about the importance of age in Tier II or above. In this study, dogs older than 6 years will be included as long they meet all the other inclusion criteria. Each dog will need to be treated with and resistant to least one ASD to be recruited. Drug-resistant patients are defined in line with the IVETF’s definition of therapeutic success Category 3 [63]. In addition, each patient must have a required seizure frequency of at least three or more seizures in the last 3 months despite properly monitored pharmacotherapy prior to enrolment. Any dog with a known cause of epilepsy (structural epilepsy) e.g. brain neoplasm, brain trauma, encephalitis and meningitis are excluded. Any acute medical or surgical condition, pregnancy, lactation, history of pancreatitis or history of chronic or acute renal, hepatic or cardiac failure would lead to the patient’s exclusion. Furthermore, dogs on drugs, diets or DS which could influence the metabolism of phenobarbitone, imepitoin or other ASD given during the trial are not included.

### Palatability

Altering the diet characteristics with oil supplementation can be at the expense of palatability and influence a dog’s eating behaviour. The mean food intake, as a percentage, can be calculated for each period and used to assess the dogs eating preference. An intake ratio (IR) can represent consumption rates. In this clinical trial successful food intake is essential. A small pilot study will be conducted in advance to ensure palatability and comparability of oils used to guarantee overall diet acceptance.

### Baseline diet

The baseline diet will be nutritionally assessed for each patient, as its composition might amplify or influence the anti-seizure properties of MCTs. This includes the individual feeding type (commercial vs. home-made; subtypes) and utilised feed components (brand, formulation and type). This information is then used to create an average main nutrient profile on a dry matter basis (MNP: crude protein, crude fat, crude ash, crude fibres, nitrogen free extracts (Nfe), moisture). For this purpose, analytical data will be requested from each company. If available, the inclusion of additional data from other relevant nutrients, such as minerals, vitamins or fatty acids, will be considered. The hypothesised anti-seizure effects from MCT oil supplementation should be then critically discussed in the light of the patients’ whole diet.

### Investigational veterinary dietary supplement

The details of the ingredients and the maintenance of each oil are stored in the Dispenser File at the monitoring site. The MCT DS used will only contain C8 and C10 in a certain ratio and therefrom allow the investigators to extend the knowledge about the role of the single medium-chain fatty acids (MCFAs) in the former publications used for cognition-enhancement in aged dogs [61] and in dogs with IE [24, 59]. Both oil DS will be commercially available. The DS will be then supplied by the Study Monitor to the Investigator and will be stored at room temperature in a locked room at each test site. The dispenser educates the owner to keep the oil administration consistent throughout the study period. From enrolment onwards, the owner is not allowed to change or give any additional foods apart from the usual diet, or other food supplementations during the study period.

### Study procedures

As aforementioned, the owner is in contact with the investigator on the following occasions with a tolerance of ±2 days: enrolment visit (−V1) before the start of the first dietary intervention period 1 (V1)); Day 45 (telephone call 1 (TC1)); Day 90 (visit 2 (V2), before the crossover and start of the second dietary intervention period II); Day 142 (telephone call 2 ((TC2)), Day 187 for study completion (visit 3 (V3)).

For each on-site study visit, the investigator completes the following tasks (a-e) to collect relevant data and monitor the clinical progress of each patient, while other parameter will only be collected depending on local conditions (f-i):

- Body weight and temperature is recorded;
- Clinical and neurological examination is performed;
- Checks the owners’ documentation of seizure occurrences by frequency, severity and subtypes using a standardised logbook.
- Visual analogue score to measure level of sedation, ataxia and quality of life;
- Blood samples for routine haematology, serum biochemistry, thyroid profile, canine pancreatic lipase (CPLI), beta-hydroxybutyrate (BHB), serum concentration of phenobarbitone and/or potassium bromide and dynamic bile acid test are taken;
- Saliva is collected in the morning of each study visit;
- Fresh urine and faeces are collected;
- Behavioural tests on anxiety and cognition are conducted;
- Gait measurements are conducted.

In order to assess the dog’s behaviour and QoL between the visits, a questionnaire is completed by the owners, at the earliest 24 h in advance of the scheduled study appointment (Additional file 3). The questionnaire consists of five existing questionnaires covering multiple aspects of behaviour, cognition, ADHD-related behaviour, appetite and QoL:

1. Canine Behavioral Assessment & Research Questionnaire (C-BARQ) [66]
2. Attention deficit hyperactivity disorder rating scale (ADHD-RS) [67]
3. Canine cognitive dysfunction rating scale (CCDR) [68]
4. Dog Obesity Risk and Appetite Questionnaire (DORA) [69]
5. Evaluation of Quality of Life in Dogs with IE (EpiQoL) [70].

All concomitant treatments and medications including vaccines, anthelmintics and drugs for parasite prophylaxis for the patient are recorded by the Investigator on each contact with the owner and is to be included in parameter analysis. Any abnormal health observation that is unfavourable and unintended and occurs after an animal has been enrolled and fed with the dietary supplement, regardless of whether it is considered to be product-related, is an adverse event. The documentation of adverse events includes the following details: a description of the adverse event; length of the adverse event recorded in days; severity of the adverse event recorded using 1 = mild, 2 = moderate, 3 = severe and 4 = serious; frequency of the adverse event measured using 1 = once, 2 = occasionally, 3 = regularly and 4 = ongoing; concomitant treatment noted as 1 = none and 2 = yes; and final outcomes consisted of 1 = resolved without further effects, 2 = resolved with further effects, 3 = unchanged, 4 = euthanasia and 5 = death. In reference to the study objectives, seizure occurrences during the study participation is not defined as an adverse event. Any concomitant treatments during the study period is recorded with details on indication, used products, given strength, doses and length of treatments. Concomitant changes of ASD medication or dosages throughout the study lead to study exclusion.

### Specification of the Study Variables (Additional file 4: Table S1)

#### General physical and neurological examination and laboratory diagnostics

A routine inter-ictal clinical and neurological examination is performed at each study visit. All above mentioned blood samples are analysed and added to the patients file. Comparison of all blood parameters between the dietary intervention periods is planned.

#### Seizure frequency, seizure days per months, inter-seizure interval

The seizure frequency is recorded by the owners in the ‘Seizure Diary’ per dietary intervention period. Short-term efficacy will be assessed over a 3-month period. Only generalised seizures will be counted toward the efficacy measurement. The primary outcome parameter for seizure frequency is ‘treatment success’: Treatment success is defined as seizure freedom for a time-span exceeding three times the longest inter-seizure interval (days) for an individual dog considering the year preceding the study and for a minimum of 3 months (time to 1st seizure) based on owners’ documentations. [63]. Consequently, patients with complete freedom of seizures or significant extension of the inter-seizure interval will be considered treatment success and treatment should thereafter be continued as open-label to assess the seizure free rate e.g. the percentage of patients with short-term or long-term freedom of seizures. Partial treatment success will only be assessed in this study for dogs, which will remain in the study for at least 3 months and have no change in treatment during this period. Partial therapeutic success is defined as a significant difference (reduction) in either: seizure frequency (seizures/month), seizure days frequency per months, seizure cluster days frequency per months, seizure severity measured by number of episodes of cluster seizures or status epilepticus during a 3-month period between the MCT and control periods. Finally, each dog will be classified as ‘seizure free’, if it experiences a 100% reduction in seizure frequency, and as a ‘MCT responder’ if it experiences at least 50% reduction in seizure frequency between both dietary periods.

#### Seizure types

Information from the standardized Seizure Diary will be used to define the type of seizures. Seizures will be classified according to the guidelines of IVETF [63]. In this study, generalised seizures are the main focus of interest, as they are better recognised by the owner and reported to their vet [71]. A generalised epileptic seizure is an epileptic seizure, which initial semiology indicates, or is consistent with, more than minimal involvement of both cerebral hemispheres. Generalised epileptic seizures are conceptualised as originating at some point within, and rapidly engaging, bilaterally distributed networks, vegetative symptoms and loss of consciousness. Focal epileptic seizures are conceptualised as originating within networks limited to one hemisphere. Cluster seizures are defined as an episode where more than one seizure occurs within a 24-h period [63]. Status epilepticus will be defined as (a) continuous seizure activity for more than 5 min or (b) two or more discrete epileptic seizures between which there is incomplete recovery of consciousness (for generalized convulsive seizures) [63].

#### Behavioural comorbidities and cognition

The conducted five questionnaires, the VAS scoring and the behavioural tests are used to evaluate the patients on neurological and behavioural changes in terms of appetite, cognition, trainability, anxiety, activity and ataxia at each visit of this trial. The questionnaires and behavioural tests will be completed during each of the three trial visits.

- Non-invasive behavioural tests:
  *Anxiety testing* [13, 72, 73]*:* Each dog will undergo two non-invasive tests to assess anxiety behaviour: (i) open field test (OFT); (ii) separation test (ST). The room setting including cameras will be standardised to ensure consistency between measurements. The recorded video clips will be retrospectively evaluated. Known anxiety-associated behaviours will be assessed in terms of frequency, intensity (likert-scale grading 1–10) and occurrence in percentage of overall time of experiment [72, 74].
  *Cognition testing*: Each dog will undergo two validated, non-invasive tests to measure different aspect of cognition (task 1: food searching, task 2: problem solving) as formerly described in dogs with and without epilepsy using the same scoring system [75, 76]. The testing environment will be prepared for both tasks in a standardised empty room with no noise, smell or light distraction.
  *Gait recording*: At each visit the gait will be video recorded and analysed retrospectively. Fifty strides from a walking dog will be assessed and then used to quantify the level of ataxia as formerly described in detail [77, 78].

(b) Questionnaires: An alteration in individual calculated scores for the questionnaires between the visits indicates improvement or deterioration of variables measured. Calculated scores are compared between dietary periods, but also grouped by MCT responder rate and seizure control in order to elucidate associations and relationships.
  i. *Canine Behavioral Assessment & Research Questionnaire (C-BARQ)* [66]*:* The cBARQ contains the following subscales used for future comparison between both dietary periods: trainability, stranger-directed fear, fear, owner-directed fear, non-social fear, separation related fear, attachment and attention seeking, chasing, excitability and touch sensitivity. Findings will be also compared to other more-targeted questionnaires, as subsequently listed.
  ii. *Attention deficit hyperactivity disorder rating scale (ADHD-RS)* [67]*:* ADHD-RS is a valid method to assess attention and activity skills in dogs. Predetermined from the 13 items, two subscales (inattention (6 items, [0–3]) and activity–impulsivity (7 items [0–3]) of the questionnaire are calculated and will be compared between both dietary periods. Multivariate analysis will also include age-, gender- and training-skills.
  iii. *Canine cognitive dysfunction rating scale (CCDR)* [68]*:* Cognitive abilities will be rated on an owner-based report using the CCDR scoring system with 13 behavioural items. The score can help identify dogs which have clinical signs seen with canine cognitive dysfunction (CCD). A score of 0–39 is classified as normal, 40–49 at risk and > 50 as CCD. Individual scores will be compared between study periods.
  iv. *Dog Obesity Risk and Appetite Questionnaire (DORA)* [69]: A questionnaire of 34 items grouped into three dog and four owner factors is applied at each study visit to get reliable, owner reported measure of the dogs eating behaviour linked to each DS. Each subfactor is rated by option scaled questions and in numerical analysis transformed as a percentage of the maximum between 0 and 100.
  v. *Evaluation of Quality of Life in Dogs with IE (EpiQoL)* [70]*:* A questionnaire of 7 themes with 36 questions compromising subunits (“Seizure severity and frequency”, “Adverse effects of anti-epileptic drug (AED)”, “Restrictions on the carer’s life”, “Frustrations over caring for a dog with IE”, “Carer distaste of AED adverse effects”, “Carer anxiety around the seizure event”, “Perceptions on rectal diazepam use”) are option scaled rated by the owner and compared in frequency.
(c) VAS: At each study visit the owner is asked to complete a VAS for ataxia, sedation and overall QoL using a line from 0 to 100 mm (0–100%). A secondary intersecting line that presented best the subjective severity should be set by the owner perpendicularly on a provided measurement line. A perpendicular line at 0 mm represents ‘asymptomatic/normal’, while at 100 mm either the dog shows ‘ataxia so severe that it is unable to walk’ or ‘sedation to the extent that it only sleeps’ or ‘QoL is so poor euthanasia is requested’. On analysis, the length from 0 to the perpendicular line will be normalised to baseline for statistical comparison into a percentage value (0–100%, negative = improvement, positive = deterioration).

#### Monitoring metabolic responses

At each study visit, blood is collected by venepuncture using standardised operation procedures for haematology, serum biochemistry, ASD serum concentrations (phenobarbital, potassium bromide), cPLI, BHB and dynamic bile acid testing. The BHB and postprandial bile acid sample is taken as one sample 2 h after feeding with oil supplement. The cortisol level in saliva will be assessed between study visits [79]. The samples will be collected at the same time in the morning of each study visit. Furthermore, serum, urine and faecal samples are collected. These samples are used to investigate the metabolome and microbiome, respectively [80].

### Data analysis

After study completion, the data will be collated, and statistical analysis will be performed using SPPS V24 (IBM Deutschland GmbH, Ehningen, Germany) and Prism® (GraphPad Software, San Diego, USA). Results will be considered significant if after correction for multiple testing the *p* value < 0.05.

For all baseline, efficacy and safety parameters, the following analysis will be performed (Additional file 4: Table S1):

- Individual values will be listed and if appropriate graphically presented per time point for each group.
- For the continuous parameters, descriptive statistics will be calculated and tabulated per time point for each group. If data are normally distributed (as ascertained via plotting histograms of relevant variables and using Kolmogorov-Smirnov test), the mean and standard error will be plotted over time for each group.
- For the categorical or discrete parameters, a frequency table will be calculated and tabulated per time point of each group. A graphical presentation over time for each group will be made by means of a bar or pie chart.

Comparisons between the DS groups are made using match-paired Student’s t-tests for parametric data and Wilcoxon matched-pairs signed rank test for non-parametric data. The relationships between two variables are analysed using Pearson’s correlation coefficient for parametric and Spearman test for non-parametric data. Optionally, further multiple comparison correction based will be applied on individual data sets. The presence of cluster seizures is compared between DS periods using the McNemar test.

Statistical analysis is planned to be a combination of univariate (UVA) and multivariate data analyses (MVA). A mixed effect model might be considered. Statistical testing will be adapted were appropriate (Additional file 4: Table S1).

### Power calculation

Our previous study did indicate that the effect of MCTs on seizure frequency can be detected with *n* = 21 dogs [24]. Similarly, another study suggested twenty-two dogs in each group being sufficient for showing significant differences between two diet groups for seizure frequency [51]. In conclusion, we aimed to recruit at least 36 dogs to reach a minimum of 22 dogs to anticipate a usual patient dropout-rate of 30%.

## Discussion

Using a crossover dietary trial approach, we will generate data to investigate the value of MCT oil supplementation as a management tool for dogs diagnosed with IE not responding to standard ASD(s). If clinically and statistically significant effects are observed between the dietary periods, MCT oil has the potential to become an easily adjustable add-on therapy for dogs with epilepsy.

This study will utilise a prospective, double-blinded randomised placebo-controlled trial with a crossover design. Considering these specific study features in the design, we do not only follow international high quality standards for clinical research, but also address potential biasing effects in clinical veterinary trials [81, 82]. The integration of a dietary crossover with placebo/control period, double-blinding and block randomisation should minimise the regression-to-the-mean, the placebo and observer effects seen in other canine epilepsy trial in veterinary medicine [83]. We are confident that this design significantly increases the accuracy and representative nature of our expected findings on the use of MCT oil as nutritional add-on management in dogs with IE.

Regression-to-the mean arises often in clinical trials when a variable is extreme on its first, but closer to the mean on its second measurement or vice versa [84–87]. In canine epilepsy trials, usually an extreme (hardest-to-treat) group is selected from an epileptic dog population based on the measurement of a particular variable. In this study, only dogs with idiopathic epilepsy TIER 2 level, classified as ‘drug-resistant’ with a poor seizure control are recruited, so that this phenomenon must be addressed in the study design. If this effect is not considered, the clinical trial or the interpretation of our obtained data can lead to incorrect conclusions, so must be then expected to be inaccurate or incorrect [88] .

The placebo effect is a well-known phenomenon in human medicine [89], however, limited information about the placebo effect exists in veterinary medicine to date [90]. Reports so far described caregiver placebo effect especially in association to chronic anti-inflammatory or analgesic treatment in small animals [91–93]. A decrease in seizure frequency of around 30% compared to baseline has been noted in different trials in canine epilepsy as potential placebo effects [83]. The occurrence of placebo effects are significantly associated with response expectancy [89]. In canine epilepsy trials, the expectation from owners is usually higher due to physiological stress, often poor seizure control and the continuous negative impact on their own QoL [70, 79, 94, 95] so that a notably subjective influence on the dog can be more likely. A general run-in period given by the recruitment process, where owners are first enrolled on a waiting list and their dog are checked on study criteria before giving the first treatment, will also support to mitigate the response expectancy.

Another impact can derive from the Hawthorne effect or also known as the observer effect. When study individuals are aware of being observed within a clinically trial, their response to the trial situation can not only modify, often falsify study variables. The Hawthorne effect can therefore also be seen as an pre-placebo expectation [96]. Alike in epilepsy research in human medicine [97], such type of reactivity should be considered as influencing factors from owners and their dogs in the design of canine epilepsy trials.

The integration of a dietary crossover in the mid of observation time, allows each participant to receive both dietary interventions. Each study object will therefore serve as its own control and reduce significant biasing effects [98]. Even though the retrospective baseline is documented, the data will be completely disregarded in most of the analysis and only applied were appropriate. This exclusion is to minimise the regression-to-the-mean and observed placebo effect in other canine epilepsy trial in veterinary medicine [83].

Keeping all involved individuals (tester, subject, owners) blind from the used dietary supplement is another attempt to eliminate bias and the expectancy from owners seeing responses in their dog’s seizure control after giving the specific DS. Not only observer, but also investigator bias might otherwise provoke incorrect observations, so that using a double-blinded design is most appropriate here. Describing the blinding methodology is important to give veterinarians the ability to assess the validity of the test results and apply in clinical environment [99] .

A core principal for clinical trials in human and veterinary medicine is the randomisation [100, 101]. The process of randomised allocation of each dog on its day of enrolment is considered in this study as being essential. The random assignment of each study subject to a predefined block (1st DS (AB), 2nd DS (BA)) reduces the impact of selection bias and increases the validity of the expected study results. Furthermore, the detailed reports about the allocation process makes it possible for practitioners to critically review the impact of bias on study results and make informed decisions regarding the treatment of their own epileptic patients [102].

Overall, using not only a crossover design with a placebo/ control period, but also a block randomisation protocol and blinding should limit all above mentioned biasing effects to a possible maximum. Acting against hypothetical, multi-sided bias by implementing described trial features, should enable us to collect and assume not only controlled, but also representative data about MCT consumption in dogs diagnosed with IE.

Dropout is often used as an outcome measure in clinical trials. But, in randomised clinical trial with epileptic patients, it appears to be higher than other clinical studies and partly explained by trial design features thus providing direction for future trial designs [103]. As a preventive measure, the total amount of patients recruited will be aimed to be increased by > 30% to ensure a sufficient number of patients that will finish the trial. Law et al. (2015) have shown an effect with 21 dogs finishing the trial. Furthermore, in another study on ketogenic diet, twenty-two dogs in each group were suggested to be sufficient for showing significant differences between two diet groups for seizure frequency [51]. Accordingly, we aimed to recruit at least 36 dogs to reach a minimum of 22 dogs factoring a usual dropout-rate of 30%.

Although study features will be implemented to prevent massive biasing effects, limitations are in any clinical trial and must be reflected critically on data analysis. One limitation of this study design is that an intention to treat analysis (ITTA) can be challenging or impossible to perform. ITTA is an approach to the analysis in which our recruited patients are evaluated as block randomised, regardless of the dietary intervention they actually received [104]. This is especially hard to realise when most or all dogs that dropout are within the first study period, as was the case in our former MCT study [24]. There is a chance that if randomisation is not carried out carefully, that results could be skewed as study dropouts will not be included in the paired statistical analysis. It is therefore of pivotal importance to document in detail (and if possible, compare statistically) the reasons why owners withdraw their dog from the study and during which study period. As aforementioned in the last study [24], the majority of the dropout dogs (7 out of 10) did not complete the first respective randomised allocated diet, hence there was no outcome measure at all for the remaining respective diet group. Using the last observation carried forward is not possible as there was no observation for the second diet.

Furthermore, utilising a statistical imputation method of the outcome measure would require profound assumptions to be made on the missing outcome. Statistical imputation of outcome assuming the MCT and placebo diet would be the same introduces bias towards the null. On the other hand, imputation assuming a difference on the probability of the outcome introduces bias towards the treatment effect. Considering that the primary aim of the study was to determine the anti-seizure efficacy of the ketogenic MCT diet, the utilising of a *per protocol* analysis method is more suitable and logical [82].

Oil supplementation for dog owners as additional treatment for general health or targeted to epilepsy management is a common practice [105]. As the clinical trial aims to prove ketogenic MCT oil supplementation as nutritional management for epileptic dogs, dietary characteristics and acceptance must be considered. As a variable, palatability is the measure of intake of a food that indicates acceptance, or the measure of preference of one food over another [55]. Food with different characteristics can alter voluntary food intake by affecting palatability, taste or flavour [56]. Excluding bias linked to palatability in the main clinical trial, the food intake ratio should be similar. The mean food intake, as a percentage, can be calculated for each period and was used to assess the dogs eating preference. An intake ratio (IR) of over 0.50 would represent equivalent consumption. If intake ratio (IR) for a feeding period was greater than 0.50 at 0.55, this would lead one to conclude that it was consumed at a greater quantity [61]. Both oils will be proven in advance in a small palatability study to be well tolerated, similar consumed and no food preference was observed. Eating time might be increased when the oil DS is given, and this may be due to ‘neophobia’ [57] - and this may take time for the dogs to habituate to the presence of a new flavour/texture in their diet [58]. In summary, a good and similar palatability without any acute gastrointestinal side effects should be concluded and monitored in the prospective dietary trial.

Canine nutrition needs to be considered for epilepsy management [60, 107–110]. Diet composition, content and some of the DSs mentioned could influence pharmacokinetic properties of ASDs and potentially modulate their efficacy [39, 61]. Owners of dogs with more severe epilepsy phenotypes, as recruited for this study, are likely to change unconsciously dietary regimes or DS due to higher levels of psychological stress [70, 79, 106], therefore owners were asked to keep diets and DS constant throughout the trial. In addition, owners were asked to daily monitor any changes in seizures, drugs used, and potential adverse events seen. Nevertheless, a balanced supply of all nutrients is routinely not checked in first opinion practice and might play a role for the interpretation of dietary effects occurring from MCT oil supplementation. We will take great care of assessing the baseline diet as aforementioned. However, each dog will have a different baseline diet, which might influence the results and could be seen as another limitation of the study.

## Additional files

Additional file 1: Pre-Study Questionnaire. Online survey designed for initial registration for the trial and to assess potential suitability of test candidate. (PDF 346 kb)

Additional file 2: SPIRIT Item Checklist (PDF 108 kb)

Additional file 3: Study Visit Questionnaire. Designed study questionnaire using five scientific validated questionnaire covering cognition, ADHD related behaviour, appetite and quality of life; Canine Behavioral Assessment & Research Questionnaire (C-BARQ, Attention deficit hyperactivity disorder Questionnaire (ADHD) [67], The canine cognitive dysfunction rating scale (CCDR), Dog Obesity Risk and Appetite Questionnaire (DORA), Evaluation of Quality of Life in Dogs with Idiopathic Epilepsy (EpiQoL) to comparing dietary effects from both dietary supplements on behaviour in dogs diagnosed with TIER2 idiopathic epilepsy. (PDF 2596 kb)

Additional file 4: **Table S1.** Study Variables: For each on-site study visit, relevant data are collected and monitored by the investigator on the clinical progress of each patient. Collected data will be later converted into predefined study variable assessing MCT as DS in comparison to the control DS. This table should provide a detailed overview, which study part will contribute to which variable by units and which statistical tests are planned to evaluate the findings on significant differences or associations. Basically, grouping will be conducted by dietary period (I vs. II), DS type (MCT vs. Control-DS), MCT responder rate (seizure frequency reduction of at least 50% or less) and seizure types (cluster seizure presence, history of status epilepticus). On evaluation of the questionnaire and behavioural test scores other relevant factors (age, weight, amount of ASD per day etc.) will be considered for grouping. (DOCX 31 kb)

## Abbreviations

ARRIVE: Animal Research: Reporting in-Vivo Experiments
ASD: Anti-seizure drug
C10: Decanoic acid
C8: Octanoic acid
CDS: Cognitive dysfunction syndrome
CIC: Clinical investigation centre
CSS: Cluster Seizure Status
DHD: Attention-deficit/hyperactivity disorder
DS: Dietary supplement
HARP: Harmonized animal research reporting principles
ICLAS: International Council for Laboratory Animal Science
IE: Idiopathic epilepsy
ITTA: Intention-to-treat analysis
IVETF: International Veterinary Epilepsy Task Force
MBW: Metabolic body weight
MCFA: Medium chain fatty acids
MCT: Medium chain triacylglyceride
ME: Metabolic energy requirement
MNP: Main nutrient profile on a dry matter basis
QoL: Quality of Life
RER: Resting energy requirement
RVC: Royal Veterinary College
