# Supplementary material for: A double-blinded randomised dietary supplement crossover trial design to investigate the short-term influence of medium chain fatty acid (MCT) supplement on canine idiopathic epilepsy: study protocol
Source: BMC Vet Res. 2019 May 30;15:181. doi: 10.1186/s12917-019-1915-8 (PMC6543566; doi:10.1186/s12917-019-1915-8)
Supplement: Supplementary file 1 — Pre-Study Questionnaire. Online survey designed for initial registration for the trial and to assess potential suitability of test candidate. (PDF 346 kb) [file 12917_2019_1915_MOESM1_ESM.pdf]

Introduction

What is this survey about?
Epilepsy is the most common chronic neurological disorder in first opinion practice for domestic dogs. Diet is increasingly recognised as having an impact upon the seizure activity and behaviour of dogs with epilepsy. We are interested in the effects of oil dietary supplements on your dog's epilepsy.

This survey will explore your dog's eligibility to enrol into our 6 month clinical trial investigating the effects of medium chain triglyceride (MCT) oil supplementation on canine epilepsy. Our decision about your dog will depend on current health status, clinical history and daily diet.

Why get involved?
Medical advances in prevention, detection and treatment of diseases in veterinary and human medicine depend on clinical trials. Clinical trials are essential to determine if a new test or treatment works and is safe. Our study is looking at the effects of a daily oil supplement added to your dog's usual diet on their seizure activity and behaviour.

What we would like from you as part of the trial
1. A complete clinical history of your dog's epilepsy.
2. Willingness to give your dog a small amount of oil twice daily and keep a seizure diary.
3. Willingness for your dog to undergo several non-invasive behavioural tests to measure their side effects.
4. You are able to make three appointments at the Queens Mother Hospital at the RVC in Hertfordshire at day 1, then 3 and 6 months later.

What do I get out of it?
1. A free veterinary check up with clinical and neurological examination at each appointment
2. Free clinical tests at each appointment including haematology, biochemistry, and serum AED levels
3. A refund of £25 x 3 to cover the costs of your drive to each appointment

We are interested in the recruitment of dogs diagnosed with epilepsy. We invite owners of dogs with epilepsy aged over 6 months, of all breeds and cross-breeds to complete this survey.

N.B. If you are selected for enrolment in the study we will contact you after completion of this survey using the contact details provided. If you are not selected we will contact you to let you know why your dog was not eligible, or if they are eligible and have been added to a waiting list.

We would be grateful if you could complete the questionnaire, which is estimated to take 20 minutes. We very much appreciate your help in this study. If you have any queries regarding the questions asked or details of the trial, please contact Dr Benjamin-Andreas Berk (bberk@rvc.ac.uk).

This study is supported by the American Kennel Club.

Data Protection and Consent

The data collected in this questionnaire will be collated and stored at the Royal Veterinary College in London (RVC). Your completion of the questionnaire indicates your consent to participate in this study. We would appreciate it, if you could give your contact details at the end of this survey, so that we can inform you about our result.

Your data will be anonymised as appropriate, and your contact details will be used only to contact you, if we need clarification of the answers to any questions forming part of this questionnaire or inform you about our decision.

Your personal information will be held and used in accordance with the Data Protection Act 1998 and will not be disclosed to any unauthorized person or body.

About You

1. Date of completion of the questionnaire

Date

MM/DD/YYYY

2. In which country do you and your dog reside?

3. In case you are eligible, at which location do you want to take part in the study?

* 4. Please select your gender

- Male
- Female
- Prefer not to say

* 5. Please select your age range

- 18-30
- 31-45
- 46-60
- 61-75
- over 76
- Prefer not to say

About your dog

* 6. Is your dog pure-bred or cross-bred?

- ◯ Pure-bred
- ◯ Cross-bred

* 7. What breed is your dog?

[ ]

If your breed is not listed please state here. If your dog is cross-bred then please state the breeds it is bred from here, if known.

[ ]

8. How old is your dog? (N.B. If your dog is less than 6 months, please do not complete this survey)

- ◯ Younger than 6 months
- ◯ Between 6 months and 12 years of age
- ◯ 12 year or older

9. What is your dogs weight in kilograms (to the nearest kg)?

[ ]

10. Does your dog have health insurance?

- ◯ Yes
- ◯ No
- ◯ I don't know

* 11. Has your dog had any of the following health problems diagnosed by a vet? (tick all that apply)

	No (Unaffected)	Yes (Mildly affected)	Yes (Moderately affected)	Yes (Severely affected)
Ear canal disease (otitis externa)	○	○	○	○
Dental disease (peridontal disease)	○	○	○	○
Arthritis (degenerative joint disease)	○	○	○	○
Gastrointestinal disease (Diarrhoea/Vomiting)	○	○	○	○
Pancreatic disease	○	○	○	○
Obesity	○	○	○	○
Injuries (traumatic injuries)	○	○	○	○
Heart problems	○	○	○	○
Canine cognitive dysfunction	○	○	○	○
Brain neoplasm, encephalitis or meningitis	○	○	○	○

Other (please explain):

12. Is your dog currently on any prescribed medication (other than routine worming/vaccinations, or anti-epileptic drugs used to control your dog's epilepsy, if your dog is affected by epilepsy)?

- ○ Yes
- ○ No

If yes, what medications? (please list brand names where possible)

13. Prehistory of renal, hepatic or cardiac failure?

- ○ Yes
- ○ No

If yes, please state when and give details

* 14. Your dog is:

- ○ Male intact
- ○ Female intact
- ○ Male neutered
- ○ Female neutered

Pregnancy, Lactation

15. Is your dog known or suspected to be pregnant or lactating or intended for breeding?

- ◯ No
- ◯ Yes
- ◯ I don't know.

Your dog's diet

16. How often do you feed your dog?

- ○ Once a day
- ○ Twice a day
- ○ Three times a day
- ○ Ad lib (free feeding/food left out)

17. Where do you usually purchase your pet food?

- ○ Supermarket
- ○ Online
- ○ Veterinary Practice
- ○ Other Petstore

18. What do you feed your dog? (select all that apply)

- ☐ Wet Food(Tinned)
- ☐ Wet Food (Pouches)
- ☐ Dry food (complete diet)
- ☐ Dry food (mixer)
- ☐ Therapeutic/Prescription/Veterinary diet
- ☐ Home cooked food
- ☐ Raw food (BARF diet)
- ☐ Vegetarian diet
- ☐ Treats (dog treats e.g. biscuits, dental sticks)
- ☐ Treats (human food e.g. table scraps)
- ☐ If you feed anything not listed in the categories above, please tell us.

19. If you feed any of the following diets please specify what it is that you feed, and in the case of commercially available feed, please type in the brand name.

Therapeutic/Prescription/ Veterinary diet

Home cooked food

Raw food

Commercially available wet or dry food

20. How did you establish your current feeding routine?

- On advice from my vet
- On advice from breeder/rescue centre
- On advice from another owner
- From information on pet food packaging
- From published information in book/magazine
- From published information online
- It is how I have always fed my dog/s
- Other

21. Do you feed your dog snacks or treats?

- Every day
- A few times a week
- A few times a month
- Never

22. Which supplements do you give your dog?

	Never	Daily	Weekly	Monthly	Less than once per month
Vitamins	◯	◯	◯	◯	◯
Glucosamine and/or chondroitin	◯	◯	◯	◯	◯
Fatty acids or oils	◯	◯	◯	◯	◯
Cod liver oil	◯	◯	◯	◯	◯
MCT oil	◯	◯	◯	◯	◯
Epitaur	◯	◯	◯	◯	◯
Milk thistle	◯	◯	◯	◯	◯
Other	◯	◯	◯	◯	◯

If Other, please specify:

Veterinary history

* 23. Has your dog ever had a seizure?

- ○ Yes
- ○ No
- ○ I don't know

* 24. Has your dog been diagnosed with epilepsy by a vet?

- ○ Yes
- ○ No

Your dog's epilepsy

* 25. How old was your dog when the first seizure occurred? (If not known, select 'Unknown')

* 26. Approximately how many seizures has your dog had in the past three months?

27. How many fits has your dog had in total? (if a precise number has not been recorded, please estimate to the nearest 10). If completely unknown, select 'Unknown'

* 28. Has your dog ever had more than one seizure in one day (24 hour period) - also known as a 'cluster seizure'?

- No - only one fit has ever occurred within 24 hours
- Yes - more than one fit has occurred within 24 hours
- I don't know

* 29. Has your dog ever experienced a seizure that lasted for longer than 5 minutes, also know as 'Status epilepticus'?

- Yes
- No
- I don't know

30. There are two types of seizures. Generalized seizures involve both sides of the brain. Common subtypes include tonic-clonic (grand mal) and absence seizures (petit mal) with spasms of the whole body, loss of consciousness. Partial (or focal) seizures are the second major seizure type. These begin in a specific area of the brain and may be contained there; these can appear as specific tics like sudden licking, swallowing or twitching, or similar.

How much percent of your dogs seizures are generalised?

Percentage:

31. Have you ever recognised focal seizures?

- Yes
- No
- I am not sure.

32. Which vet did you see to discuss your dog's seizures?

- ◯ My local vet(s) only
- ◯ My local vet(s) AND a Neurology Specialist

* 33. Which of the following tests were carried out by your vet(s) to diagnose epilepsy? Tick all that apply

- ☐ Blood tests
- ☐ Urine tests
- ☐ MRI scan of the brain
- ☐ CT scan of the brain
- ☐ Lumbar puncture/ Cerebrospinal fluid (CSF) analysis
- ☐ Tests were done, but I am uncertain of the details

* 34. What was the diagnosis for your dog's seizures?

- ◯ Idiopathic epilepsy (i.e. no cause found)
- ◯ Other causes e.g. a brain tumour, liver problems, a stroke, meningitis

35. Does your dog currently receive any medication to treat their epilepsy?

- ◯ Yes
- ◯ No

Epilepsy treatment - Antiepiletic medication

* 36. Does your dog CURRENTLY receive any of the following epilepsy medications? (Tick as many as apply)

- [ ] Imepitoin (Pexion)
- [ ] Phenobarbital (Epiphen, Phenoleptil)
- [ ] Potassium Bromide (Epilease, Libromide)
- [ ] Levetiracetam (Keppra)
- [ ] Zonisamide
- [ ] Gabapentin
- [ ] Diazepam
- [ ] Pregablin
- [ ] Chlorazepate
- [ ] Felbamate
- [ ] Phenytoin
- [ ] Lamotrigine
- [ ] Tiagabine
- [ ] Vigabatrin

37. What doses of medication is your dog currently receiving to control the seizures?

Please copy the information written on the bottles for all medication including medication name, medication strength, dose and frequency

EXAMPLE
Medication: Epiphen, 60mg tablet, 2 tablets, twice daily

Medication ______

Medication ______

Medication ______

Medication ______

Medication ______

Medication ______

38. When did your dog's current epilepsy medication regime commence (N.B. If your dog is on ONE medication, the date this was started. If your dog is on MORE THAN ONE medication, the date that their most recent medication first, then the second and the third one.)

Date

DD/MM/YYYY

Date

DD/MM/YYYY

Date

DD/MM/YYYY

39. How many seizures per month (on average) did your dog have BEFORE this date?

40. How many seizures per month (on average) has your dog had SINCE this date?

Your dog's seizures

41. Think about the most common type of seizure your dog has. Which signs does your dog show during this type of seizure? Select ALL that apply

- [ ] Falls on the floor
- [ ] Body goes floppy/limp
- [ ] Body goes stiff
- [ ] Running/paddling movements
- [ ] Facial twitches
- [ ] Chewing movements
- [ ] Rhythmic blinking
- [ ] Head shaking
- [ ] Twitching of one leg
- [ ] Excessive salivation/drooling
- [ ] Vomits
- [ ] Urinates
- [ ] Defecates
- [ ] Pupils dilate
- [ ] Fearful/anxious behaviour
- [ ] Fly snapping
- [ ] Doesn't respond to my voice
- [ ] Cannot look me in the eye
- [ ] Other (please specify)

42. How long do your dog's most common type of seizures last on average? (in minutes)

Thank you and data information

Thank you very much for your time and effort in answering this questionnaire!

Your data will be used only for the current study and the results will be published in a veterinary journal. Summaries of the results will be made available on the RVC website.

43. If you want to be a team for our 6 months clinical trial, please provide your contact data below:

Forename, Surname:	
Email address:	
First three digits of your post code:	
Telephone number:	
Mobile phone number:	
Telephone availability (Day, time):	
