# Supplementary material for: A double-blinded randomised dietary supplement crossover trial design to investigate the short-term influence of medium chain fatty acid (MCT) supplement on canine idiopathic epilepsy: study protocol
Source: BMC Vet Res. 2019 May 30;15:181. doi: 10.1186/s12917-019-1915-8 (PMC6543566; doi:10.1186/s12917-019-1915-8)
Supplement: Supplementary file 3 — Study Visit Questionnaire. Designed study questionnaire using five scientific validated questionnaire covering cognition, ADHD related behaviour, appetite and quality of life; Canine Behavioral Assessment & Research Questionnaire (C-BARQ, Attention deficit hyperactivity disorder Questionnaire (ADHD) [67], The canine cognitive dysfunction rating scale (CCDR), Dog Obesity Risk and Appetite Questionnaire (DORA), Evaluation of Quality of Life in Dogs with Idiopathic Epilepsy (EpiQoL) to comparing dietary effects from both dietary supplements on behaviour in dogs diagnosed with TIER2 idiopathic epilepsy. (PDF 2596 kb) [file 12917_2019_1915_MOESM3_ESM.pdf]

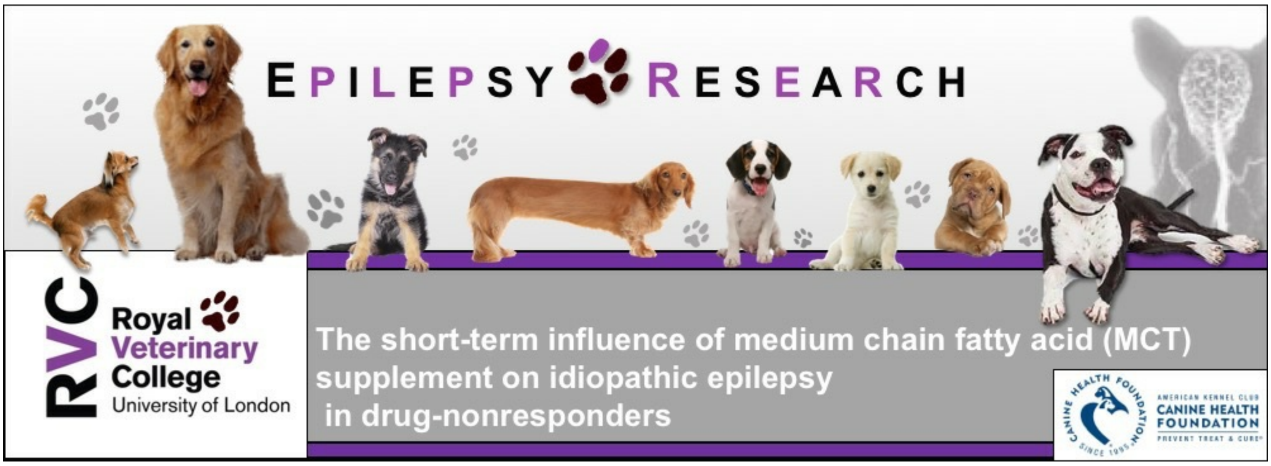

MCT Study - Visit 1 - Questionnaire

Introduction

Welcome to our study visit!

First of all, we want to thank you for being a part of our clinical study on the effects of dietary supplements on canine epilepsy. We are very pleased that you have decided to support us in our challenge of developing effective non-drug treatments for canine epilepsy.

Clinical trials are an essential part of this process, and our study is looking at the effects of an oil, given daily as a supplement to your dogs diet, on the seizure activity and behaviour of your dog. In order to evaluate the efficacy of this oil as a treatment, we are interested in detailed information about your dog's seizure characteristics, behaviour, activity, gait and appetite.

As part of this study, we invite you now to complete this survey exploring the following five characteristics of your dog:

1. Temperament and Behaviour
2. Activity and Attention
3. Ageing Behaviour
4. Appetite
5. Quality of Life

We would be grateful if you could complete each questionnaire once. For your answers to be of use you have to complete the full list of questionnaires, which is estimated to take around 1 hour.

We very much appreciate your help in this study. If you have any queries regarding the questions, do not hesitate to contact Dr Benjamin-Andreas Berk (bberk@rvc.ac.uk) directly.

Data Protection and Consent

The data collected in this questionnaire will be collated and stored at the Royal Veterinary College in London (RVC). Your completion of the questionnaire indicates your consent to participate in this study. Your data will be anonymized as appropriate, and your contact details will be used only to contact you, if

we need clarification of the answers to any questions forming part of this questionnaire or inform you about our decision.

Your personal information will be held and used in accordance with the Data Protection Act 1998 and will not be disclosed to any unauthorized person or body. We are happy to send you an electronic summary of the study results (by email only) at the end of the official clinical study.

Only the principal investigators in this study (Prof Holger Volk, Dr Benjamin-Andreas Berk, Dr Rowena Packer) will have access to the data on the returned questionnaires. The anonymous results of the will be published on the RVC website and in a journal. N.B. The study was approved by the Clinical Research Ethical Review Board (CRERB) and ethical approval has been granted (URN 2016 1558).

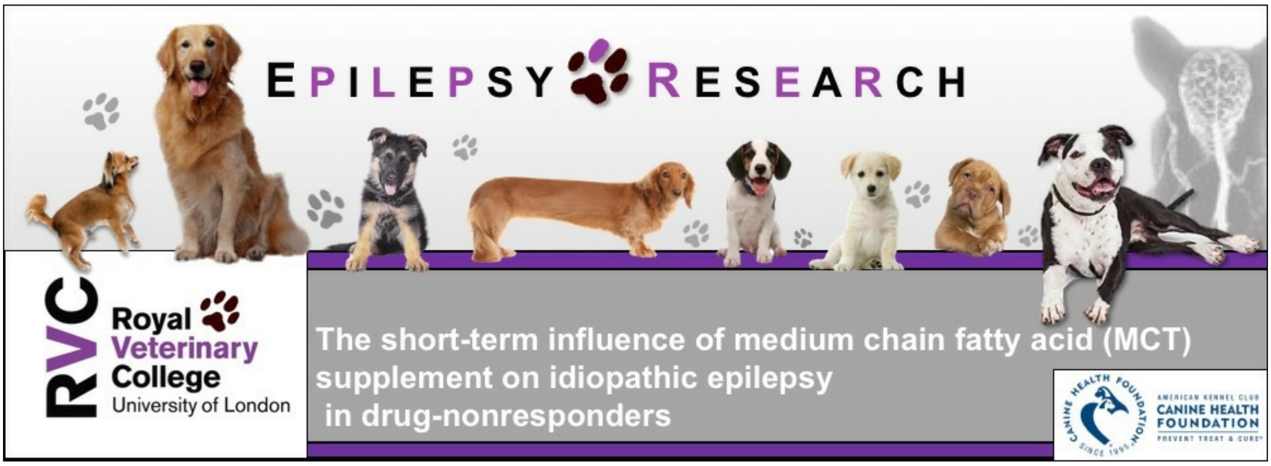

MCT Study - Visit 1 - Questionnaire

About You

*** 1. Please type in your full name, plus your dogs name:**

Forename [ ]

Surname [ ]

Dog name [ ]

2. Which Investigation Centre are you completing the study at?

[ ]

*** 3. What is your dog's Study Number?**

[ ]

4. Which visit is this today?

[ ]

*** 5. Date of completion of the questionnaire**

Date

MM/DD/YYYY

6. In which country do you and your dog reside?

[ ]

[ ]

*** 7. Please select your gender**

- ◯ Male
- ◯ Female
- ◯ Prefer not to say

*** 8. Please select your age range**

- ◯ 18-30
- ◯ 31-45
- ◯ 46-60
- ◯ 61-75
- ◯ over 76
- ◯ Prefer not to say

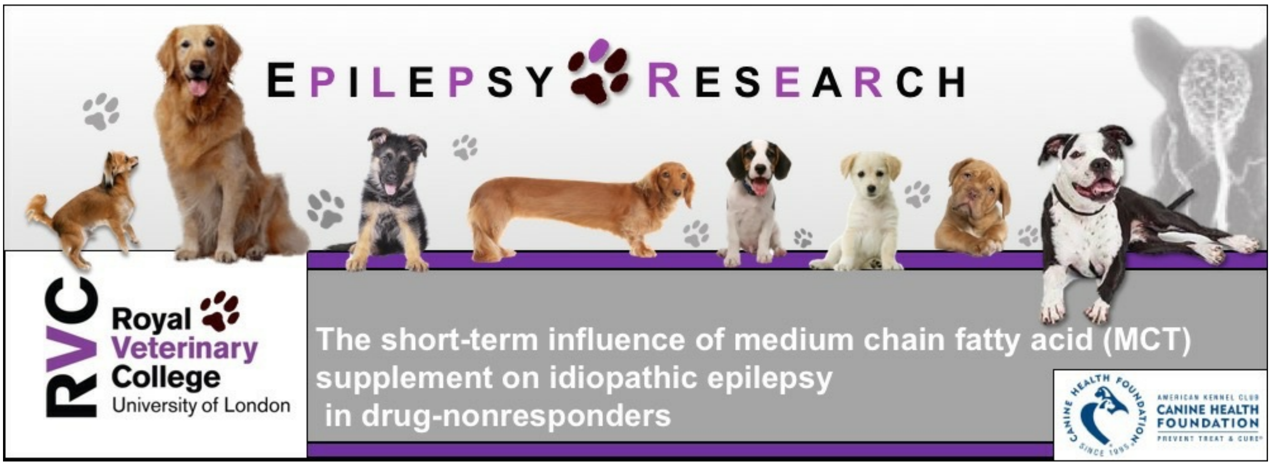

MCT Study - Visit 1 - Questionnaire

Your dog

*** 9. Is your dog pure-bred or cross-bred?**

- ○ Pure-bred
- ○ Cross-bred

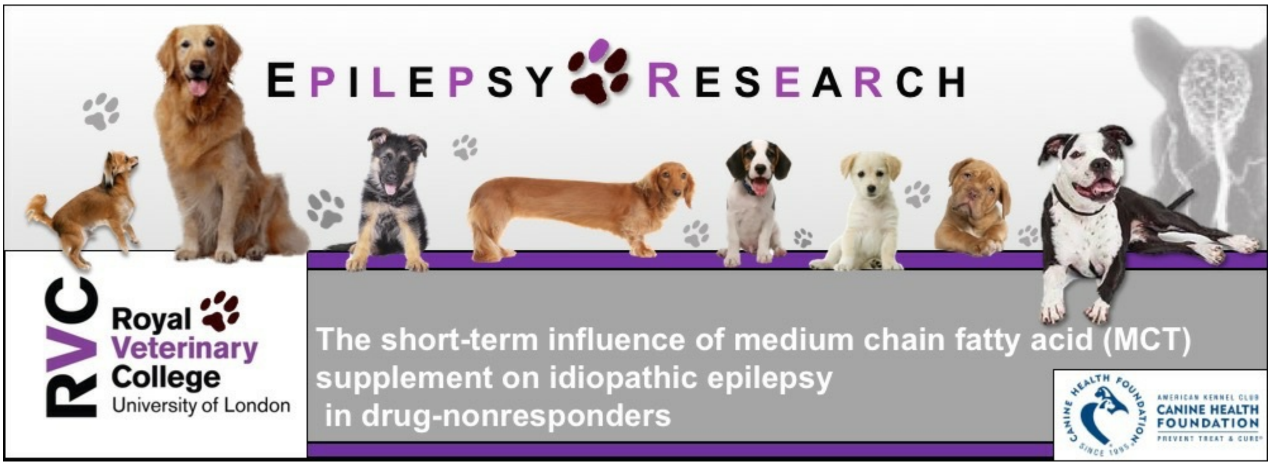

MCT Study - Visit 1 - Questionnaire

Your dog 2

10. If your dog is pure-bred, is your dog registered with a breed association or kennel club in your country?

- ◯ Yes
- ◯ No
- ◯ I don't know
- ◯ Other (please specify)

____

*** 11. What breed is your dog?**

____

If your breed is not listed please state here. If your dog is cross-bred then please state the breeds it is bred from here, if known.

____

*** 12. What sex is your dog**

- ◯ Female entire
- ◯ Female neutered
- ◯ Male entire
- ◯ Male neutered
- ◯ Chemically neutered.

13. If your dog is neutered, at what age was it neutered?

*** 14. How old is your dog, please state as XX years XX months?**

15. How old was your dog when you bought/rehomed them?

16. What is your dogs weight in kilograms (to the nearest kg)?

17. Is your dog currently on any prescribed medication other than routine worming/vaccinations, or anti-epileptic drugs used to control your dog's epilepsy?

- Yes
- No

If yes, what medications? (please list brand names where possible)

18. Does your dog have health insurance?

- Yes
- No
- I don't know

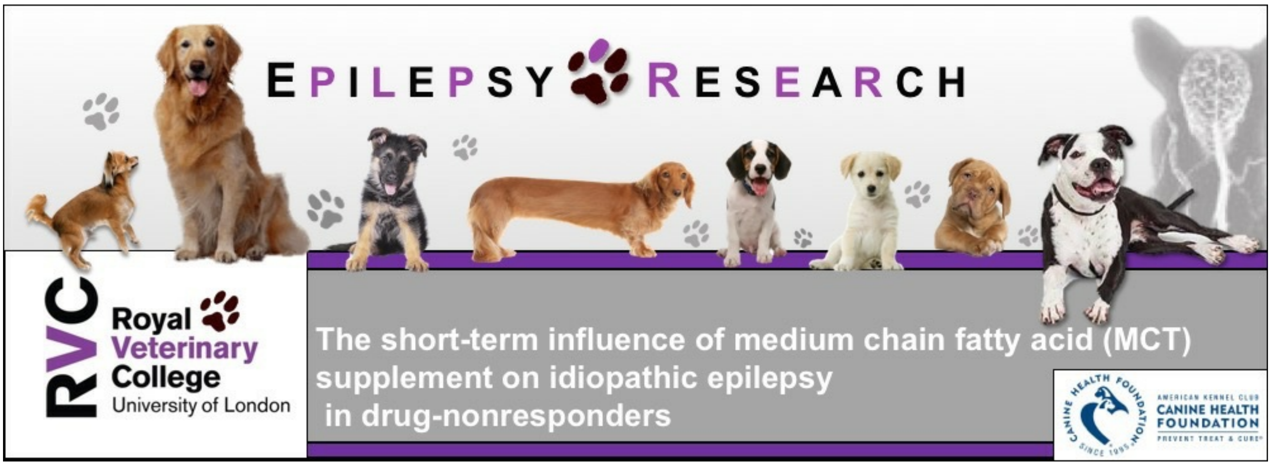

MCT Study - Visit 1 - Questionnaire

Veterinary history

*** 19. Has your dog ever had a seizure?**

- ◯ Yes
- ◯ No
- ◯ I don't know

*** 20. Has your dog been diagnosed with epilepsy by a vet?**

- ◯ Yes
- ◯ No

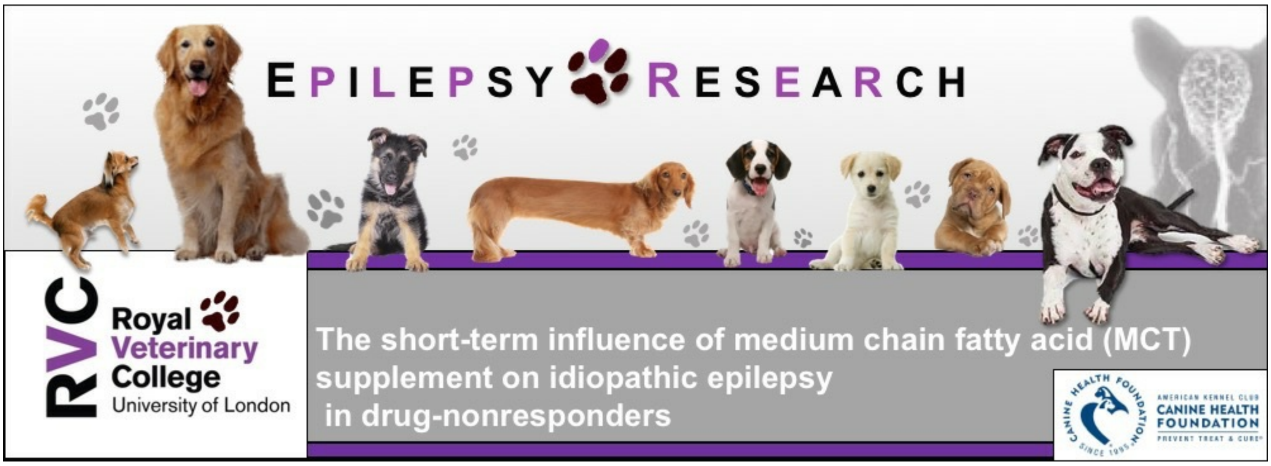

MCT Study - Visit 1 - Questionnaire

Epilepsy diagnosis

*** 21. To your knowledge, has your dog had 2 or more seizures (if only 2, they were at least 24 hours apart)?**

- ◯ Yes
- ◯ No

*** 22. Was your dog's first seizure between the ages of 6 months and 6 years?**

- ◯ Yes
- ◯ No

*** 23. Has a vet carried out blood and urine tests on your dog and found no identifiable cause for his/her seizures?**

- ◯ Yes
- ◯ No

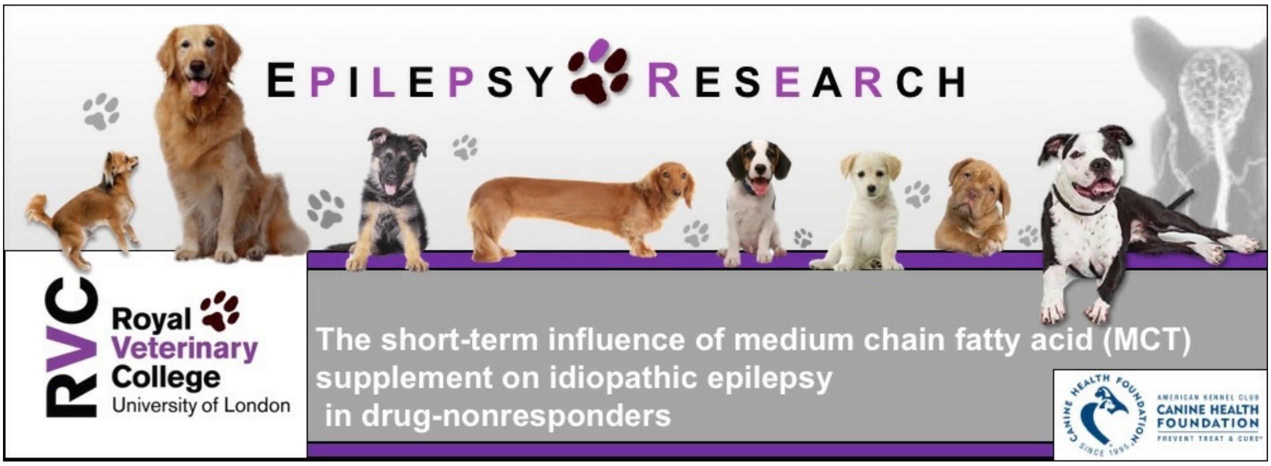

MCT Study - Visit 1 - Questionnaire

Your dog's epilepsy

*** 24. How old was your dog when the first seizure occurred? (If not known, select 'Unknown')**

[ ]

25. How many fits has your dog had in total? (if a precise number has not been recorded, please estimate to the nearest 10). If completely unknown, select 'Unknown'

[ ]

26. Which vet did you see to discuss your dog's seizures?

- ◯ My local vet(s) only
- ◯ My local vet(s) AND a Neurology Specialist
- ◯ Please specify - Name, Clinic/ Practice:

[ ]

*** 27. Which of the following tests were carried out by your vet(s) to diagnose epilepsy? Tick all that apply**

- ☐ Blood tests (haematology, biochemistry, bile acids etc.)
- ☐ Urine tests
- ☐ MRI scan of the brain
- ☐ CT scan of the brain
- ☐ Lumbar puncture/ Cerebrospinal fluid (CSF) analysis
- ☐ Tests were done, but I am uncertain of the details

* **28. What was the diagnosis for your dog's seizures?**

- ◯ Idiopathic epilepsy (i.e. no cause found)
- ◯ Other causes e.g. a brain tumour, liver problems, a stroke, meningitis

29. When did your dog last have a seizure?

- ◯ Within the last 24 hours
- ◯ Within the last 7 days
- ◯ 8-14 days ago
- ◯ 15-21 days ago
- ◯ 22-28 days ago
- ◯ 1-3 months ago
- ◯ 4-6 months ago
- ◯ 7-12 months ago
- ◯ Over 12 months ago
- ◯ I don't know

30. Approximately how many seizures has your dog had in the past three months?

[▲▼]

* **31. Has your dog ever had more than one seizure in one day (24 hour period) - also known as a 'cluster seizure'?**

- ◯ No - only one fit has ever occurred within 24 hours
- ◯ Yes - more than one fit has occurred within 24 hours
- ◯ I don't know

* **32. Has your dog ever experienced a seizure that lasted for longer than 5 minutes?**

- ◯ Yes
- ◯ No
- ◯ I don't know

* **33. Has your dog ever had two or more seizures in a row without fully regaining consciousness in between them?**

- ◯ Yes
- ◯ No
- ◯ I don't know

34. Does your dog currently receive any medication to treat their epilepsy?

- ◯ Yes
- ◯ No

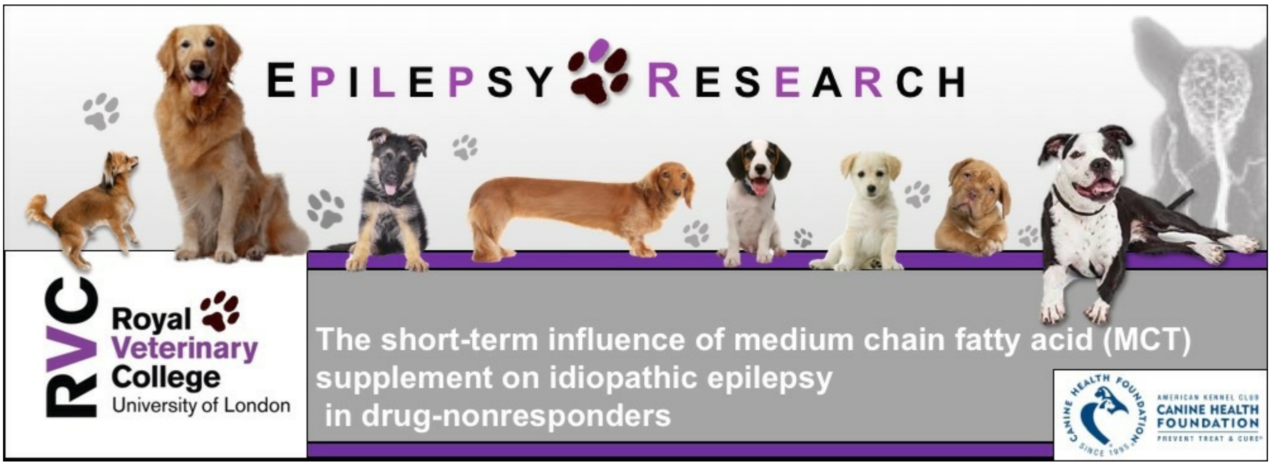

MCT Study - Visit 1 - Questionnaire

Epilepsy treatment

*** 35. Does your dog CURRENTLY receive any of the following epilepsy medications? (Tick as many as apply)**

- [ ] Imepitoin (Pexion)
- [ ] Phenobarbital (Epiphen, Phenoleptil)
- [ ] Potassium Bromide (Epilease, Libromide)
- [ ] Levetiracetam (Keppra)
- [ ] Zonisamide
- [ ] Gabapentin
- [ ] Diazepam
- [ ] Pregablin
- [ ] Chlorazepate
- [ ] Felbamate
- [ ] Phenytoin
- [ ] Lamotrigine
- [ ] Tiagabine
- [ ] Vigabatrin

36. What doses of medication is your dog currently receiving to control the seizures?

Please copy the information written on the bottles for all medication including medication name, medication strength, dose and frequency. Start with the most recent and end at the oldest medication he got.

EXAMPLE
Medication: Epiphen, 60mg tablet, 2 tablets, twice daily

Medication

Medication

Medication

Medication

Medication

Medication

Medication

37. When did your dog's current epilepsy medication regime commence (N.B. If your dog is on ONE medication, the date this was started. If your dog is on MORE THAN ONE medication, the date that their <u>most recent</u> medication was added first, then the second one.

Date
MM/DD/YYYY

Date
MM/DD/YYYY

Date
MM/DD/YYYY

Date
MM/DD/YYYY

Date
MM/DD/YYYY

38. How many seizures per month (on average) did your dog have BEFORE this date?

39. How many seizures per month (on average) has your dog had SINCE this date?

40. Does your dog experience side effects from their anti-epileptic medication, and if so, how severe are they? If your dog did not show this side effect, please tick ‘not present’. Please tick ALL that apply.

	Not present	Very Mild	Mild	A moderate amount	Severe	Very Severe
Eating more / would like to eat more	○	○	○	○	○	○
Gaining weight	○	○	○	○	○	○
Drinking more	○	○	○	○	○	○
Urinating more	○	○	○	○	○	○
Sleeping more than before	○	○	○	○	○	○
Wobbly / not coordinated when walking	○	○	○	○	○	○
Restlessness / pacing	○	○	○	○	○	○
Itchiness or skin rash	○	○	○	○	○	○
Vomiting	○	○	○	○	○	○
Diarrhoea	○	○	○	○	○	○
Coughing	○	○	○	○	○	○

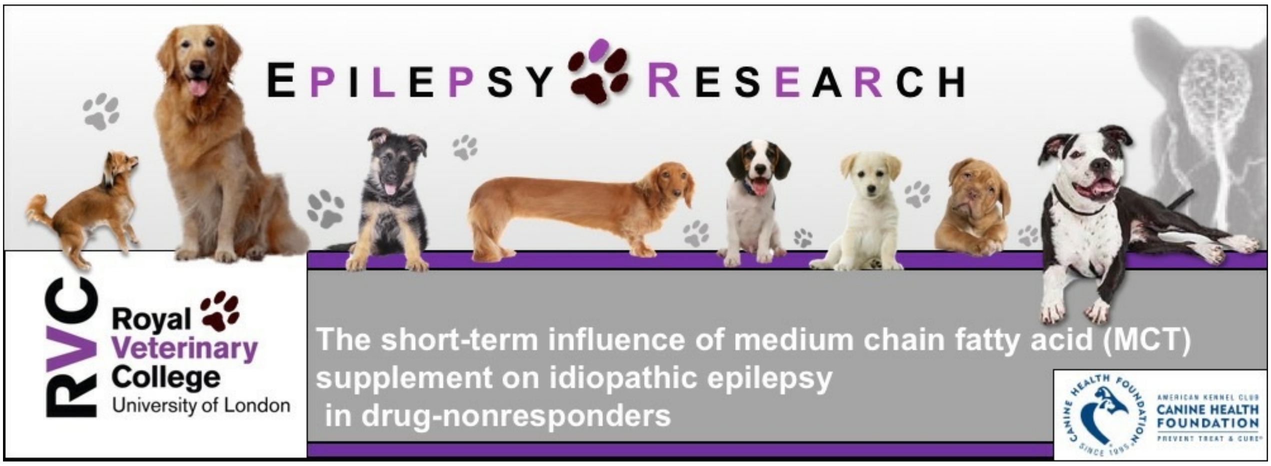

MCT Study - Visit 1 - Questionnaire

Your dog's seizures

41. Think about the <u>most common</u> type of seizure your dog has. Which signs does your dog show during this type of seizure? Select ALL that apply

- ☐ Falls on the floor
- ☐ Rhythmic blinking
- ☐ Defecates
- ☐ Body goes floppy/limp
- ☐ Head shaking
- ☐ Pupils dilate
- ☐ Body goes stiff
- ☐ Twitching of one leg
- ☐ Fearful/anxious behaviour
- ☐ Running/paddling movements
- ☐ Excessive salivation/drooling
- ☐ Fly snapping
- ☐ Facial twitches
- ☐ Vomits
- ☐ Doesn't respond to my voice
- ☐ Chewing movements
- ☐ Urinates
- ☐ Cannot look me in the eye
- ☐ Other (please specify)

____

42. How long do your dog's <u>most common</u> type of seizures last on average? (in minutes)

____

43. On a scale of 1 (very mild) to 7 (very severe), please rate how severe your dog's <u>most common</u> type of seizures are on average

	1 - Not at all severe	2 - Very mild	3 - Mild	4 - Moderate	5 - Severe	6 - Very Severe	7 - Could not be worse	N/A
Severity	○	○	○	○	○	○	○	○

44. What signs does your dog show while recovering from their <u>most common</u> type of seizure? Select ALL that apply. If your dog is normal immediately after a seizure select 'None of the above'

My dog is:

- ☐ wobbly/ ataxic
- ☐ disorientated
- ☐ staring
- ☐ blind /can't see very well
- ☐ sleepy
- ☐ fearful /scared
- ☐ aggressive
- ☐ restless /pacing
- ☐ sniffing
- ☐ hungry
- ☐ thirsty
- ☐ clingy/wants more attention than normal
- ☐ Normal - none of the above

45. How long does it take for your dog to COMPLETELY recover from their <u>most common</u> seizure type?

My dog:

- ○ is usually completely back to normal within minutes after the fit
- ○ is usually completely back to normal within 1-6 hours after the fit
- ○ is usually completely back to normal within 6 hours after the fit
- ○ is usually completely back to normal within 24 hours after the fit
- ○ is usually completely back to normal within 48 hours after the fit
- ○ usually takes more than 48 hours to recover completely

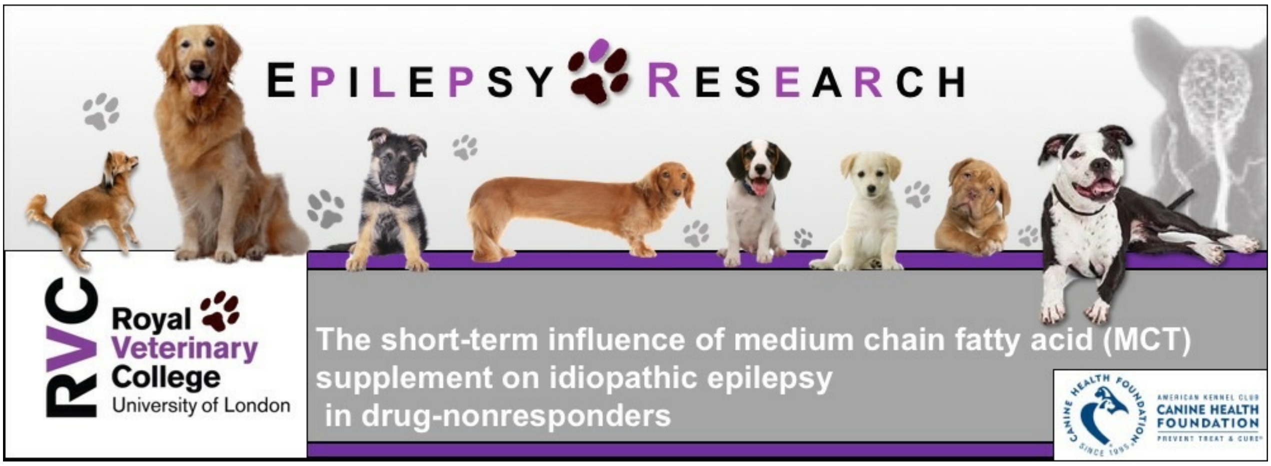

MCT Study - Visit 1 - Questionnaire

Your dog's behaviour: Temperament and Behaviour - 1

*** 46. How often does your dog do the following?**

	Never	Seldom	Sometimes	Usually	Always
Returns immediately when called while off leash	○	○	○	○	○
Obeys a sit command immediately	○	○	○	○	○
Obeys a stay command immediately	○	○	○	○	○
Will fetch or attempt to fetch sticks, balls and other objects	○	○	○	○	○
Seems to attend to or listen closely to everything you say or does	○	○	○	○	○
Is slow to respond to correction or punishment	○	○	○	○	○
Is slow to learn new tricks or tasks	○	○	○	○	○
Is easily distracted by interesting sights, sounds or smells	○	○	○	○	○

*** 47. Does your dog ever react aggressively to the following situations?**

	Never	Seldom	Sometimes	Usually	Always
When approached directly by an unfamiliar male adult while being walked or exercised on a lead	○	○	○	○	○
When approached directly by an unfamiliar female adult while being walked or exercised on a lead	○	○	○	○	○
When approached directly by an unfamiliar child while being walked or exercised on a lead	○	○	○	○	○
Toward unfamiliar persons approaching the dog while it is in the owner’s car	○	○	○	○	○
When an unfamiliar person approaches the owner or a member of the owner’s family at home	○	○	○	○	○
When an unfamiliar person approaches the owner or a member of the owner’s family away from home	○	○	○	○	○
When mailmen or other delivery workers approach the home	○	○	○	○	○
When strangers walk past the home while the dog is in the garden	○	○	○	○	○
When joggers, cyclists, roller skaters, or skateboarders pass the home while the dog is in the garden	○	○	○	○	○

	Never	Seldom	Sometimes	Usually	Always
Toward unfamiliar persons visiting the home	○	○	○	○	○
When approached directly by an unfamiliar dog of the same or larger size	○	○	○	○	○
When approached directly by an unfamiliar dog of a smaller size	○	○	○	○	○

*** 48. Does your dog ever respond aggressively to the following situations?**

	Never	Seldom	Sometimes	Usually	Always
When verbally corrected or punished by a member of the household	○	○	○	○	○
When toys, bones, or other objects are taken away by a member of the household	○	○	○	○	○
When bathed or groomed by a member of the household	○	○	○	○	○
When approached directly by a member of the household while it is eating	○	○	○	○	○
When food is taken away by a member of the household	○	○	○	○	○
When stared at directly by a member of the household	○	○	○	○	○
When stepped over by a member of the household	○	○	○	○	○
When a member of the household retrieves food or objects stolen by the dog	○	○	○	○	○

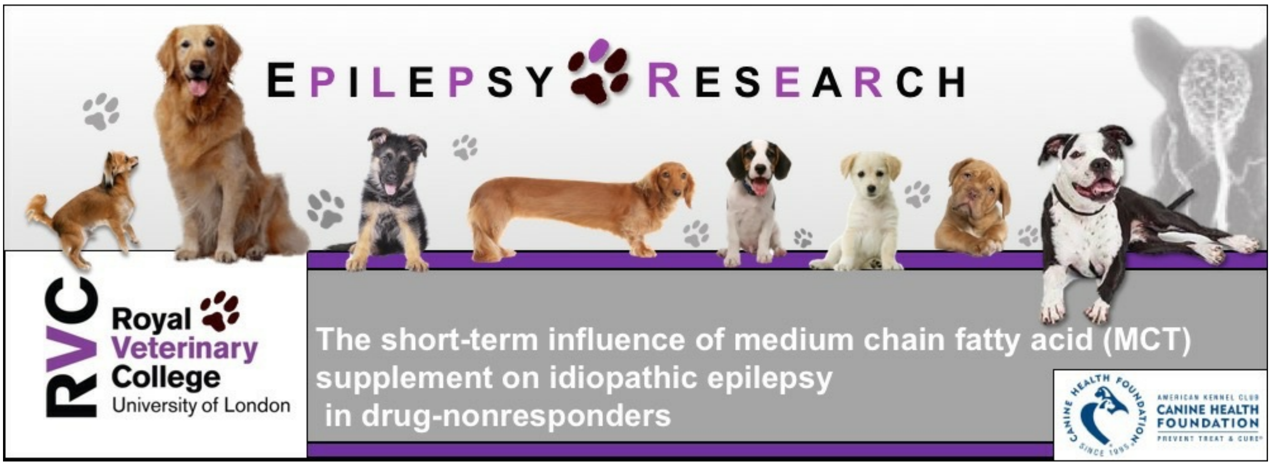

MCT Study - Visit 1 - Questionnaire

Your dog's behaviour: Temperament and Behaviour - 2

*** 49. Please assess the following situations and determine how likely your dog is to respond in a fearful or anxious way:**

	Never	Seldom	Sometimes	Usually	Always
When approached directly by an unfamiliar male adult while away from the home	○	○	○	○	○
When approached directly by an unfamiliar female adult while away from the home	○	○	○	○	○
When approached directly by an unfamiliar child while away from the home	○	○	○	○	○
When unfamiliar persons visit the home	○	○	○	○	○
In response to sudden or loud noises	○	○	○	○	○
In heavy traffic	○	○	○	○	○
In response to strange or unfamiliar objects on or near the pavement	○	○	○	○	○
During thunderstorms	○	○	○	○	○
When first exposed to unfamiliar situations	○	○	○	○	○
In response to wind or wind-blown objects	○	○	○	○	○

*** 50. Does your dog display the following behaviour?**

	Never	Seldom	Sometimes	Usually	Always
Shaking, shivering, or trembling when left or about to be left on its own	○	○	○	○	○
Excessive salivation when left or about to be left on its own	○	○	○	○	○
Restlessness, agitation, or pacing when left or about to be left on its own	○	○	○	○	○
Whining when left or about to be left on its own	○	○	○	○	○
Barking when left or about to be left on its own	○	○	○	○	○
Howling when left or about to be left on its own	○	○	○	○	○
Chewing or scratching at doors, floor, windows, and curtains when left or about to be left on its own	○	○	○	○	○
Loss of appetite when left or about to be left on its own	○	○	○	○	○

*** 51. Which category best describes your dog’s behaviour?**

	Never	Seldom	Sometimes	Usually	Always
Displays a strong attachment for a particular member of the household	○	○	○	○	○
Tends to follow a member of household from room to room about the house	○	○	○	○	○
Tends to sit close to or in contact with a member of the household when that individual is sitting down	○	○	○	○	○

	Never	Seldom	Sometimes	Usually	Always
Tends to nudge, nuzzle, or paw a member of the household for attention when that individual is sitting down.	○	○	○	○	○
Becomes agitated when a member of the household shows affection for another person	○	○	○	○	○
Becomes agitated when a member of the household shows affection for another dog or animal	○	○	○	○	○
Acts aggressively toward cats, squirrels, and other animals entering its garden	○	○	○	○	○
Chases cats if given the chance	○	○	○	○	○
Chases birds if given the chance	○	○	○	○	○
Chases squirrels and other small animals if given the chance	○	○	○	○	○
Tends to nudge, nuzzle, or paw a member of the household for attention when that individual is sitting down.	○	○	○	○	○

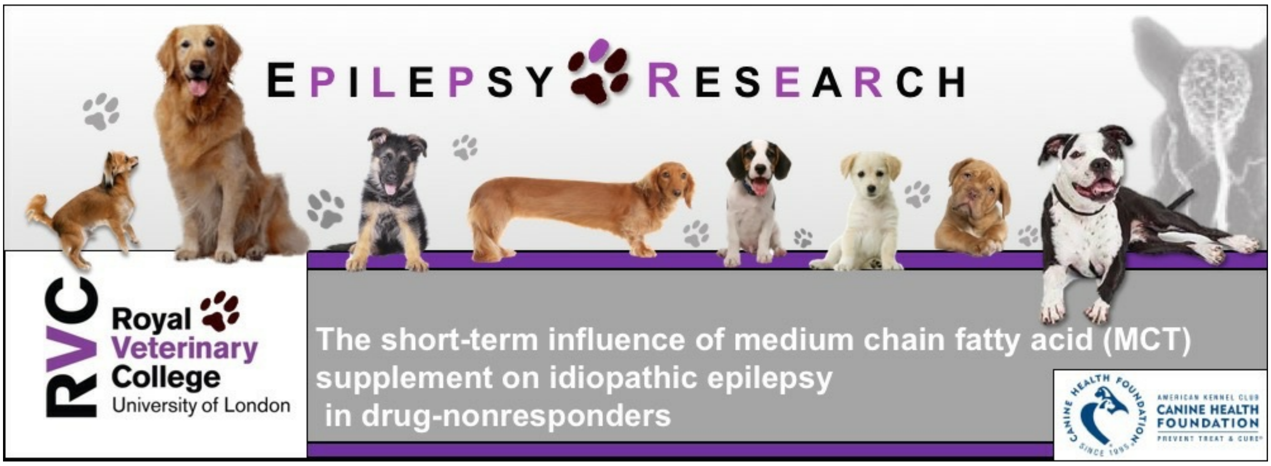

MCT Study - Visit 1 - Questionnaire

Your dog's behaviour: Temperament and Behaviour - 3

* 52. Does your dog respond in a highly excitable way to the following situations?

	Never	Seldom	Sometimes	Usually	Always
When a member of the household returns home after a brief absence	○	○	○	○	○
When playing with a member of the household	○	○	○	○	○
When the doorbell rings	○	○	○	○	○
Just before being taken for a walk	○	○	○	○	○
Just before being taken on a car trip	○	○	○	○	○
When visitors arrive at its home	○	○	○	○	○

*** 53. Does your dog react in a fearful or anxious way to the following situations?**

	Never	Seldom	Sometimes	Usually	Always
When examined or treated by a veterinarian	◯	◯	◯	◯	◯
When having its claws clipped by a household member	◯	◯	◯	◯	◯
When groomed or bathed by a household member	◯	◯	◯	◯	◯

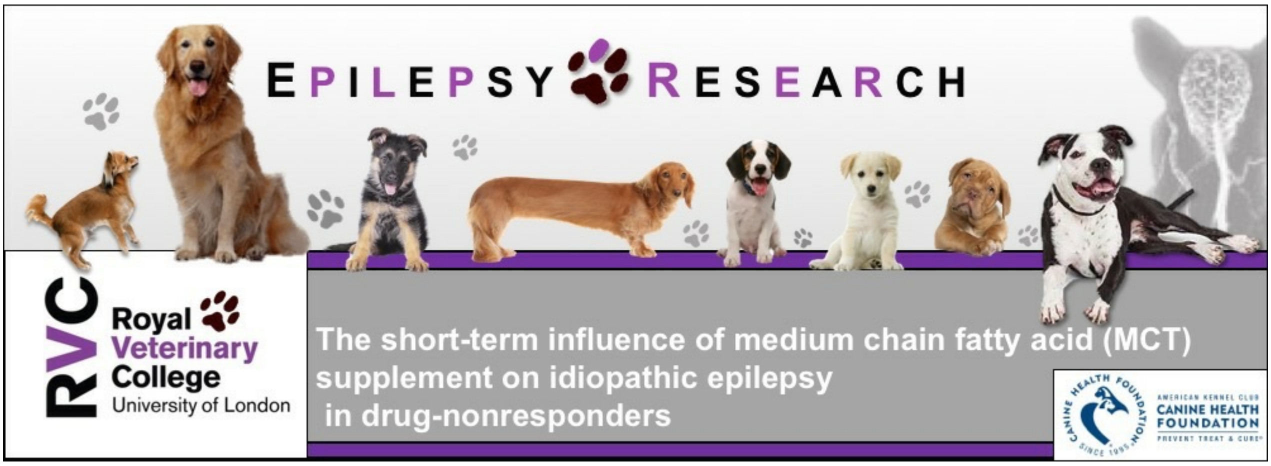

MCT Study - Visit 1 - Questionnaire

Your dog's behaviour: Activity and Attention

*** 54. Please indicate how often each statement is true for your dog.**

	Never	Sometimes	Often	Very often
Your dog has difficult time learning, because it is careless or other things can easily attract its attention.	○	○	○	○
It is easy to attract its attention, but it loses its interest soon.	○	○	○	○
It is difficult for it to concentrate on a task or play.	○	○	○	○
It leaves from its place when it should stay.	○	○	○	○
It can not be quiet, it can not be easily calmed.	○	○	○	○
It fidgets all the time.	○	○	○	○
It seems that it does not listen even if it knows that someone is speaking to it.	○	○	○	○
It is excessive, difficult to control, if it lunges, it is hard to hold back.	○	○	○	○
It would always play and run.	○	○	○	○
It solves simple tasks easily, but it often has difficulties with complicate tasks, even of to know them and has practiced them often.	○	○	○	○
It is likely to react hastily and that is why it is failing tasks.	○	○	○	○
Its attention can be easily distracted.	○	○	○	○
It can not wait as in it has no self-control.	○	○	○	○

55. Training qualification the dog has had:
Please indicate whether the dog passed an exam or just participated in training.

- ○ Trained at home
- ○ No training
- ○ Agility
- ○ Hunting
- ○ Schutzhund
- ○ Obedience
- ○ Other (please specify)

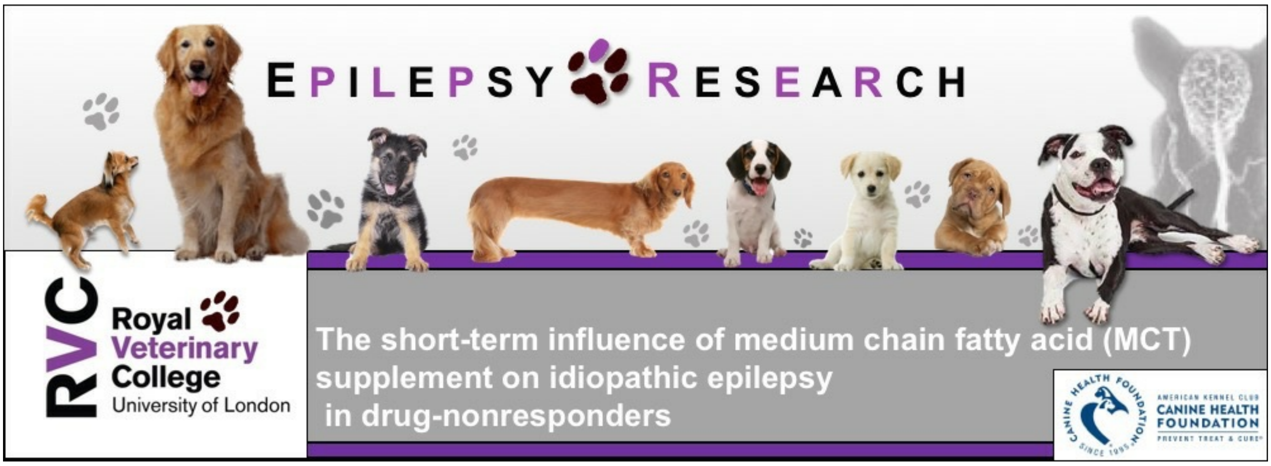

MCT Study - Visit 1 - Questionnaire

Your dog's behaviour: Ageing Behaviour

*** 56. How often does your dog show the following behaviours?**

	Never	Once a month	Once a week	Once a day	> Once a day
How often does your dog pace up and down, walk in circles and/or wander with no direction or purpose?	◯	◯	◯	◯	◯
How often does your dog stare blankly at the walls or floor?	◯	◯	◯	◯	◯
How often does your dog get stuck behind objects and is unable to get around?	◯	◯	◯	◯	◯
How often does your dog fail to recognise familiar people or pets?	◯	◯	◯	◯	◯
How often does your dog walk into walls or doors?	◯	◯	◯	◯	◯
How often does your dog walk away while, or avoid, being patted?	◯	◯	◯	◯	◯

*** 57. Please rate the following question based on how your dog behave:**

	Never	1-30% of times	31-60% of times	61-99% of times	Always
How often does your dog have difficulty finding food dropped on the floor?	◯	◯	◯	◯	◯

58. Compared with 6 months ago, does your dog show the following behaviours?

	Much less	Slightly less	The same	Slightly more	Much more
Compared with 6 months ago, does your dog now pace up and down, walk in circles and/or wander with no direction or purpose?	◯	◯	◯	◯	◯
Compared with 6 months ago, does your dog now stare blankly at the walls or floor?	◯	◯	◯	◯	◯
Compared with 6 months ago, does your dog urinate or defecate in an area it has previously kept clean (if your dog has never house-soiled, tick ‘the same’)?	◯	◯	◯	◯	◯
Compared with 6 months ago, does your dog have difficulty finding food dropped on the floor?	◯	◯	◯	◯	◯
Compare with 6 months ago, does your dog fail to recognise familiar people or pets?	◯	◯	◯	◯	◯
Compared with 6 months ago, is the amount of time your dog spends active?	◯	◯	◯	◯	◯

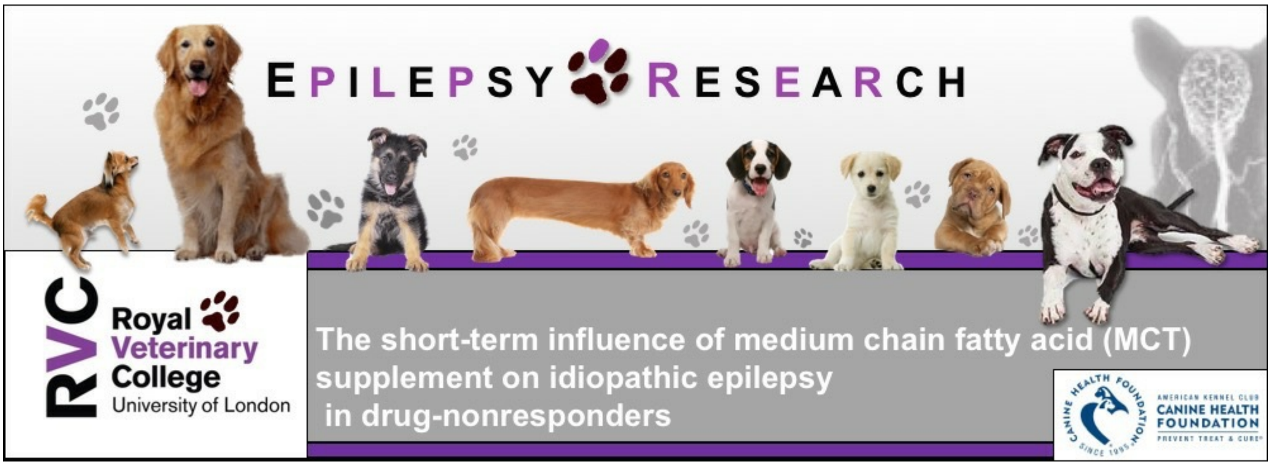

MCT Study - Visit 1 - Questionnaire

Your dog's behaviour:
Appetite

*** 59. Please score the following situations with your dog:**

	Never	Rarely	Sometimes	Often	Always
My dog gets excited when there is food around	○	○	○	○	○
My dog spends most of his/her walks off the lead	○	○	○	○	○
My dog gets human leftovers in his/her food bowl	○	○	○	○	○
My dog hangs around for titbits even if there is not much chance of getting them	○	○	○	○	○
My dog is choosy about which titbits he eats	○	○	○	○	○
My dog hangs around when I am preparing or eating human food	○	○	○	○	○
My dog will turn down food if s/he is not hungry	○	○	○	○	○
My dog finishes a meal straight away	○	○	○	○	○
My dog inspects unfamiliar foods before deciding whether to eat them	○	○	○	○	○
My dog runs around a lot	○	○	○	○	○
After a meal my dog is still interested in eating	○	○	○	○	○
My dog takes his/her time to eat a meal	○	○	○	○	○
My dog eats titbits straight away	○	○	○	○	○
My dog gets bits of human food when we are eating	○	○	○	○	○

60. Please score the following situations with your dog:

	Not at all true	Somewhat true	Mainly true	Definitely true
My dog would eat anything.	○	○	○	○
My dog is very fit.	○	○	○	○
My dog often gets human food.	○	○	○	○
My dog gets an upset tummy on some foods.	○	○	○	○
I think my dog could do with losing some weight.	○	○	○	○
My dog's walks are mostly on the lead.	○	○	○	○
I restrict my dog's exercise because of veterinary advice.	○	○	○	○
I alter the food my dog gets in order to control his/her weight.	○	○	○	○
My dog seems to be hungry all the time.	○	○	○	○
My dog's walks involve a lot of energetic play or chasing.	○	○	○	○
I am careful about my dog's weight.	○	○	○	○
My dog has a sensitive stomach.	○	○	○	○
My dog is very greedy.	○	○	○	○
My dog regularly sees the vet for health problems.	○	○	○	○
I am happy with my dog's weight.	○	○	○	○
I weigh or measure how much food I give my dog.	○	○	○	○
I am careful to regulate the exercise my dog gets in order to keep him/her slim.	○	○	○	○

	Not at all true	Somewhat true	Mainly true	Definitely true
My dog gets a lot of exercise.	○	○	○	○
My dog often gets tummy upsets.	○	○	○	○
My dog gets no food at human mealtimes.	○	○	○	○
My dog would eat non-food objects like stones, toys or socks.	○	○	○	○

61. How greedy is your dog? Please grade your dog on a scale of 0 - 10, where 0 is not at all greedy and 10 is as greedy as a dog can get.

62. Use this box to tell us anything about your dog's eating behaviour, weight, or management that you think is important.

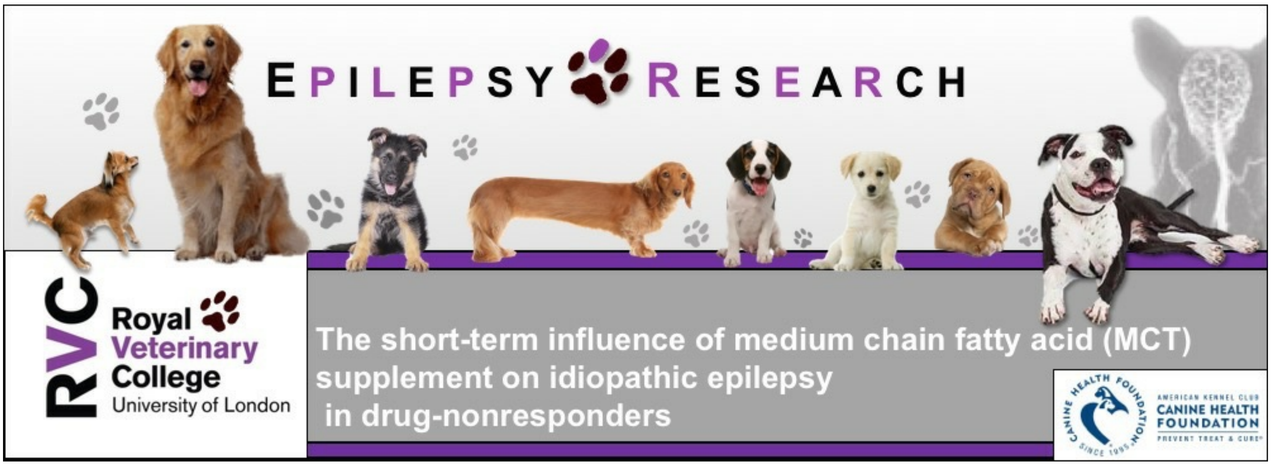

MCT Study - Visit 1 - Questionnaire

Your dog's behaviour: Quality of Life - 1

*** 63. Seizure severity and frequency**

	Strongly agree	Partly agree	Agree	Partly disagree	Strongly disagree
In the last 3 months, the frequency of the fits in my dog was acceptable:	○	○	○	○	○
In the last 3 months, the severity of the fits in my dog was acceptable:	○	○	○	○	○
In the last 3 months, overall, the fits in my dog are managed successfully:	○	○	○	○	○
Overall, how severe were your dog’s fits in the past 3 months?	○	○	○	○	○

64. Adverse effects of anitepileptic drugs

	Very mild	Mild	Moderat	Severe	Very severe
In the past 3 months, the adverse effects of the medication to control the fits in my dog were acceptable	○	○	○	○	○
In the past 3 months, how severe was the following overall adverse effect:	○	○	○	○	○
Eating more/would like to eat more	○	○	○	○	○
Gaining weight	○	○	○	○	○
Drinking more	○	○	○	○	○
Urinating more	○	○	○	○	○
Sleeping more than before	○	○	○	○	○
Wobbly/not coordinated when walking	○	○	○	○	○
Restlessness/pacing	○	○	○	○	○
Itchiness or skin rash	○	○	○	○	○
Vomiting	○	○	○	○	○
Diarrhea	○	○	○	○	○
Coughing	○	○	○	○	○

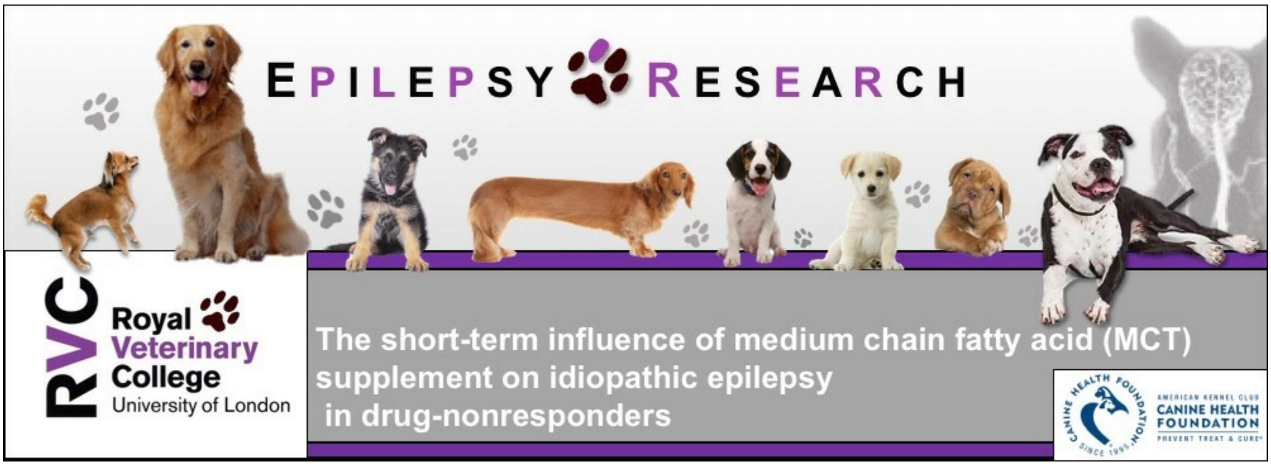

MCT Study - Visit 1 - Questionnaire

Your dog's behaviour: Quality of Life - 2

*** 65. Restrictions on the carer's life (related to caring for a dog with idopathic epilepsy):**

	Never	Rarely	Sometimes	Often	Very often
In the past 3 months, how often did you feel that your dog's epilepsy caused conflict with your work, education, or day-to-day activities?	○	○	○	○	○
In the past 3 months, how often did you feel that your dog's epilepsy limited your social life?	○	○	○	○	○
In the past 3 months, how often did you feel that your dog's epilepsy limited your independence?	○	○	○	○	○

66. Frustrations over caring for a dog with idiopathic epilepsy:

	Not at all bothersome	Somewhat bothersome	Bothersome	Mainly bothersome	Extremely bothersome
My limitations in work, education or day-to-day activities because of my dog's fits	○	○	○	○	○
My social limitations because of my dog's fits	○	○	○	○	○
Overall, the limitations on my life caring for my epileptic dog	○	○	○	○	○

67. Owner distaste of antiepileptic drugs adverse effects:

	Not at all bothersome	Somewhat bothersome	Bothersome	Mainly bothersome	Extremely bothersome
How bothersome are the physical effects of the medication on my dog?	○	○	○	○	○
How bothersome are the mental effects of the medication on my dog?	○	○	○	○	○

68. Owner distaste of AED adverse effects - In the past 3 months, how much did you dislike the following adverse effects?

	Not at all	Slightly	Moderately	Very	A lot
Eating more/would like to eat more	○	○	○	○	○
Gaining weight	○	○	○	○	○
Drinking more	○	○	○	○	○
Urinating more	○	○	○	○	○
Sleeping more than before	○	○	○	○	○
Wobbly/not coordinated when walking	○	○	○	○	○
Restlessness/pacing	○	○	○	○	○
Coughing	○	○	○	○	○

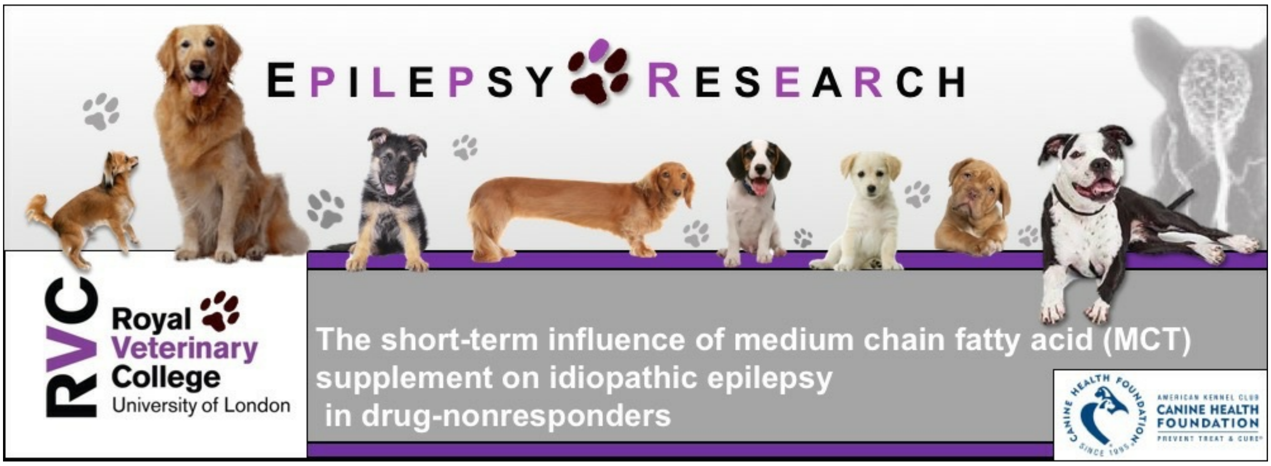

MCT Study - Visit 1 - Questionnaire

Your dog's behaviour: Quality of Life - 3

*** 69. Carer anxiety around the seizure event (and its effects on the dog):**

	Strongly agree	Partly agree	Agree	Partly disagree	Strongly disagree
In the last 3 months, I worried about the frequency of the fits in my dog					
In the last 3 months, I worried about the severity of the fits in my dog					

70. Perceptions of rectal diazepam use:

	Never	Rarely	Sometimes	Often	Very often
Have you ever been uncertain when to give rectal diazepam?					
Have you ever been worried how much or how often you are supposed to give rectal diazepam?					

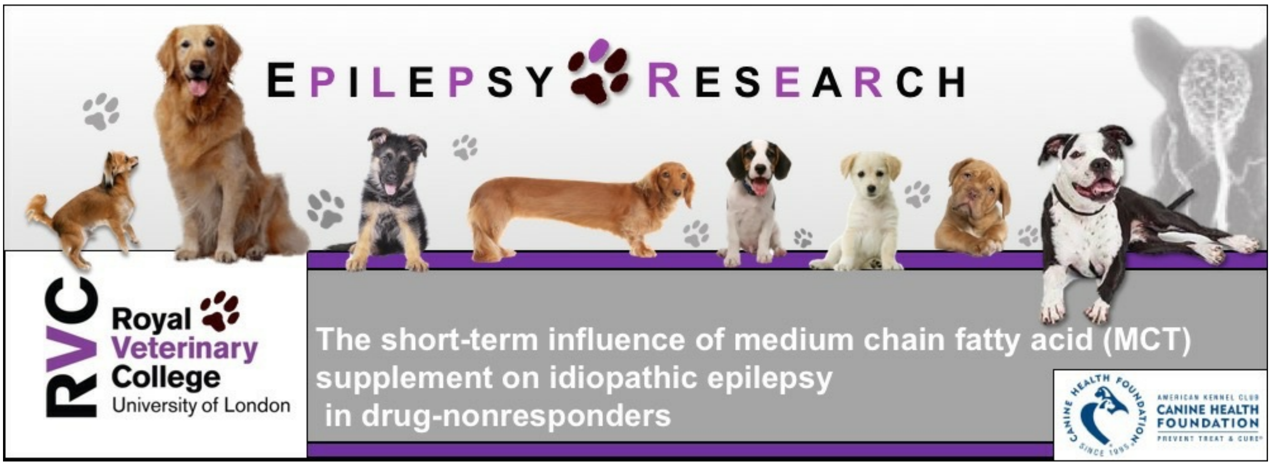

MCT Study - Visit 1 - Questionnaire

Thank you and data information

Thank you very much for your time and effort in answering the big questionnaire. Please **save the date of your next visit in your diary**! We look forward to seeing you at the next visit!

Your data will be used only for the current study and the results will be published in a veterinary journal. Summaries of the results will be made available on the RVC website.

If you have any questions, please contact Dr. Ben Berk by bberk@rvc.ac.uk.
We wish you and your dog the best for the first dietary intervention period.
