# Supplementary material for: A double-blinded randomised dietary supplement crossover trial design to investigate the short-term influence of medium chain fatty acid (MCT) supplement on canine idiopathic epilepsy: study protocol
Source: BMC Vet Res. 2019 May 30;15:181. doi: 10.1186/s12917-019-1915-8 (PMC6543566; doi:10.1186/s12917-019-1915-8)
Supplement: Supplementary file 4 — Table S1. Study Variables: For each on-site study visit, relevant data are collected and monitored by the investigator on the clinical progress of each patient. Collected data will be later converted into predefined study variable assessing MCT as DS in comparison to the control DS. This table should provide a detailed overview, which study part will contribute to which variable by units and which statistical tests are planned to evaluate the findings on significant differences or associations. Basically, grouping will be conducted by dietary period (I vs. II), DS type (MCT vs. Control-DS), MCT responder rate (seizure frequency reduction of at least 50% or less) and seizure types (cluster seizure presence, history of status epilepticus). On evaluation of the questionnaire and behavioural test scores other relevant factors (age, weight, amount of ASD per day etc.) will be considered for grouping. (DOCX 31 kb) [file 12917_2019_1915_MOESM4_ESM.docx]

**Table 1 -** **Study Variables:** For each on-site study visit, relevant data are collected and monitored by the investigator on the clinical progress of each patient. Collected data will be converted into predefined study variables assessing the effect of MCT as a DS in comparison to the control DS. This table provides a detailed overview of each planned variable and corresponding statistical tests. Grouped comparison of variables will include dietary period (I vs. II), DS type (MCT vs. Control-DS), MCT responder rate (seizure frequency reduction of at least 50%, or less) and epilepsy phenotype (cluster seizure presence, history of status epileptics). [N = value/ amount, U = Unit, U/l = Unit per l, % = percent]

| **Measuring points for all variables*** | Study Visit 1* | Telephone Call 1 (TC1) | Study Visit 2* (+ Wash-Out) | Telephone Call 2 (TC2) | Study Visit 3* |
| --- | --- | --- | --- | --- | --- |
|  |  |  |  |  |  |
| **Study Day** | 0, day of enrolement | 45±2 | 90±2 (+ 7 days) | 142±2 | 187±2 |

| **Study variable** | | **Variable type** | | **Variable type** | **Subtype** | **Statistical tests** | |  |
| --- | --- | --- | --- | --- | --- | --- | --- | --- |
|  |  | **Outcome** | **Covariate** |  |  | **Non-parametric** | **Parametric** |  |
|  | |  |  |  |  |  |  |  |
| **Demographics** | |  |  |  |  |  |  |  |
| Body weight (kg) | X |  | kg | continuous | Wilcoxon-signed rank test | Paired t-Test |  |  |
| Age (months) |  | X | months | continuous | Wilcoxon-signed rank test | Paired t-Test |  |  |
| Body temperature (°C) |  | X | °C | continuous | Wilcoxon-signed rank test | Paired t-Test |  |  |
|  |  |  |  |  |  |  |  |  |
| **Examination** | |  |  |  |  |  |  |  |
| Clinical | X | X | abnormal/normal | categorical | Spearman C | Chi-Square X^2^ Test |  |  |
| Neurological: mentation, level of consciousness, posture, coordination, gait, cranial nerves, limb nerves, sensory exam | X | X | abnormal/normal, grading (0 = absent, 1 = decreased, 2 = normal, 3 = increased, 4 = clonus) | categorical | Spearman C | Chi-Square X^2^Test |  |  |
| Owner reported sedation, ataxia and quality of life via visual analogue scale (VAS) | X | X | 0 – 100, change in % | continuous / categorical | Wilcoxon-signed rank test; Spearman C | Paired t-Test; Chi-Square X^2^Test |  |  |
|  |  |  |  |  |  |  |  |  |
| **Seizure Data** | |  |  |  |  |  |  |  |
| Seizure frequency | X |  | N per months, study phase, change in % | continuous | Wilcoxon-signed rank test | Paired t-Test |  |  |
| Seizure days | X |  | N per months, study phase, change in % | continuous | Wilcoxon-signed rank test | Paired t-Test |  |  |
| Seizure types | X |  | presence/ absence | categorical | Spearman C | Chi-Square X^2^Test |  |  |
| Cluster seizure days/ occurrence | X |  | N per months, positive/ negative, change in % | continuous / categorical | Wilcoxon-signed rank test; Spearman C; McNemar-Test | Paired t-Test; Chi-Square X^2^Test; McNemar-Test |  |  |
| Status epilepticus | X |  | N per months, positive/ negative | continuous / categorical | Wilcoxon-signed rank test; Spearman C, McNemar-Test | Paired t-Test; Chi-Square X^2^Test; McNemar-Test |  |  |
| Inter-seizure interval | X |  | In days, months, change in % | continuous / categorical | Wilcoxon-signed rank test; Spearman C | Paired t-Test; Chi-Square X^2^Test |  |  |
| Responder rate | X |  | % of seizure reduction, responders vs. non-responders | continuous / categorical | Wilcoxon-signed rank test; Spearman C | Paired t-Test; Chi-Square X^2^Test |  |  |
|  |  |  |  |  |  |  |  |  |
| **Laboratory Diagnostics** | |  |  |  |  |  |  |  |
| Routine haematology |  | X | relative, absolute values | continuous | Wilcoxon-signed rank test | Paired t-Test |  |  |
| Serum biochemistry |  | X | mmol/l, U/l, mg/dl | continuous | Wilcoxon-signed rank test | Paired t-Test |  |  |
| Canine pancreatic lipase activity |  | X | U/l | continuous | Wilcoxon-signed rank test | Paired t-Test |  |  |
| Beta-hydroxybutyrate | X |  | mmol/l | continuous | Wilcoxon-signed rank test | Paired t-Test |  |  |
| Pre- and postprandial bile acid serum concentration | X |  | mmol/l | continuous | Wilcoxon-signed rank test | Paired t-Test |  |  |
| Drug serum concentrations |  | X | ug/ml, mg/dl, mg/l | continuous | Wilcoxon-signed rank test | Paired t-Test |  |  |
|  | | |  |  |  |  |  |  |
| **Other physiological variables** | | |  |  |  |  |  |  |
| Saliva cortisol levels | X |  | mg/ml | continuous | Wilcoxon-signed rank test | Paired t-Test |  |  |
| Urine analysis | X |  | positive vs. negative; specific gravity | continuous / categorical | Wilcoxon-signed rank test; Spearman C | Paired t-Test; Chi-Square X^2^Test |  |  |
| Neurotransmitter concentration in urine: serotonin, dopamine, epinephrine, norepinephrine, glutamate, gamma-aminobutyric acid, histamine, glycine, phenethylamine | X |  | mmol/l, ug/ml, ug/g | continuous | Wilcoxon-signed rank test | Paired t-Test |  |  |
| Metabolome | X |  | Relative/absolute metabolite concentration  mmol/l, ug/ml | continuous | Wilcoxon-signed rank test | Paired t-Test |  |  |
| Microbiome | X |  | Operational Taxonomic Units (OTUs); physiological/ pathological | continuous / categorical | Wilcoxon-signed rank test; Spearman C | Paired t-Test; Chi-Square X^2^Test |  |  |
|  |  |  |  |  |  |  |  |  |
| **Practical Behaviour Tests** | | |  |  |  |  |  |  |
| Anxiety testing: open-field testing, separation testing [13, 70, 71] | X |  | trait occurrence, frequency, duration | continuous / categorical | Wilcoxon-signed rank test; Spearman C | Paired t-Test; Chi-Square X^2^Test |  |  |
| Cognition testing: food searching task, problem solving task [73,74] | X |  | Ability to locate food reward: scores (1-4) [76] | continuous / categorical | Wilcoxon-signed rank test; Spearman C | Paired t-Test; Chi-Square X^2^Test |  |  |
| Gait measurement [75,76] | X |  | six gait parameters, coefficient of variation [78] | Continuous/ categorical | N=2, Wilcoxon-signed rank test; N>3, Kruskal-Wallis-Test; Spearman C | N=2, Paired t-Test; N >2, ANOVA, Chi-Square X^2^Test |  |  |
|  |  |  |  |  |  |  |  |  |
| **Questionnaires** | | |  |  |  |  |  |  |
| C-BARQ (all subscales) [64] | X |  | score, option-scaled rating (1-4) | Continuous/ categorical | N=2, Wilcoxon-signed rank test; N>3, Kruskal-Wallis-Test; Spearman C | N=2, Paired t-Test; N >2, ANOVA, Chi-Square X^2^Test |  |  |
| ADHD-RS [65] | X |  | Inattention, activity-impulsivity subscales, separate scores: 0-3 | Continuous/ categorical | N=2, Wilcoxon-signed rank test; N>3, Kruskal-Wallis-Test; Spearman C | N=2, Paired t-Test; N >2, ANOVA, Chi-Square X^2^Test |  |  |
| CCDR-Score [66] | X |  | score: 0-80 | Continuous | N=2, Wilcoxon-signed rank test; N>3, Kruskal-Wallis-Test; Spearman C | N=2, Paired t-Test; N >2, ANOVA, Chi-Square X^2^Test |  |  |
| DORA –Score [67] | X |  | 34 items, score: 1-100 | Continuous/ categorical | N=2, Wilcoxon-signed rank test; N>3, Kruskal-Wallis-Test; Spearman C | N=2, Paired t-Test; N >2, ANOVA, Chi-Square X^2^Test |  |  |
| EpiQol items [68] | X |  | 7 themes, 36 items; option-scaled rating (1-5) | Continuous/ categorical | N=2, Wilcoxon-signed rank test; N>3, Kruskal-Wallis-Test; Spearman C | N=2, Paired t-Test; N >2, ANOVA, Chi-Square X^2^Test |  |  |
